# Advancing Patient-Centered Care: A Nationwide Analysis of Hospital Efficiency and Morbidity Using Innovative Propensity Score Techniques

**DOI:** 10.7759/cureus.76370

**Published:** 2024-12-25

**Authors:** Samy Allam

**Affiliations:** 1 Quality and Health Data Integrity, Arrowhead Regional Medical Center, Colton, USA; 2 Medical Education, California University of Science and Medicine, Colton, USA

**Keywords:** ai and machine learning, electronic health record (ehr), geometric mean length of stay, health-care equity, health quality, key performance indicators, length-of-stay, patient care outcomes, patient’s satisfaction, propensity score-matching analysis

## Abstract

Introduction

The patient-centered care model emphasizes patient autonomy in recovery, acknowledging each individual's unique journey. Despite challenges in the healthcare system, this model has gained traction nationwide. Advances in healthcare technology have highlighted obstacles to independent decision-making. This study addresses these issues by emphasizing the need for consistent access to health information, which is crucial for empowering patients. We aim to proactively identify information gaps and propose new insights for better data precision and synchronization protocols. Our analysis of nationwide hospital length of stay (LOS) data demonstrates data-driven interventions tailored to patients’ needs, aiming to improve the hospital experience and reduce care fragmentation.

Methods

We examined the complex nature of hospital LOS and various variables across nationwide healthcare settings using CMS data from 2011 to 2021. To enhance our national findings, we incorporated a local perspective by analyzing LOS data from Arrowhead Regional Medical Center (ARMC) and its associated diagnosis-related groups (DRGs). We employed a propensity score to adjust for variables and proactively drive realistic predictions of hospital outcomes. This methodological approach emphasizes the importance of using tools that can be scaled from localized settings to a broader national context. Furthermore, our study highlights the critical need for continuous quality assessment of hospital LOS. This includes measuring LOS and developing innovative tools capable of predicting, analyzing, intervening, and prompting actions based on insights gained from data analysis. The study aims to achieve several core objectives by integrating these components: enhancing patient empowerment through improved communication, refining LOS assessment through innovative techniques, and developing predictive tools to inform clinical practice and policy. Ultimately, this research contributes to a more patient-centered approach to managing inpatient care, improving patient outcomes and satisfaction.

Results

Our study aspires to transform three pivotal domains that can enhance patient autonomy, optimize hospital recovery, and elevate the overall experience. First, the cost of care reveals that prolonged hospital stays and escalating expenses are often linked to more severe health consequences. Second, our analysis uncovers the intricate relationship between hospital outcomes, such as mortality and readmissions, showing that shorter hospital stays can diminish patients' risk of complications. However, we must tread carefully, as this approach may lead to premature discharges. Lastly, providers can gain more precise insights into these interconnected outcomes by leveraging data tools such as propensity scores. We advocate for the dissolution of care fragmentation through robust health information exchange (HIE), and the adoption of innovative strategies such as blockchain and advanced machine learning (ML) techniques that rise to contemporary medicine and adapt to the growing patient needs.

## Introduction

A patient-centered care model inspires a transformative healthcare journey by placing the patient at its core, honoring their autonomy, values, and preferences as they navigate their path to recovery. This approach serves as both a theoretical concept and a practical guide for healthcare professionals, researchers, and policymakers to follow daily. Despite this commitment, many patients need help in maintaining autonomy due to their limited capacity or understanding of medical conditions and the complexities of the current healthcare system, health information enterprises, intertwined insurance policies, covered hospital stay durations, and associated managed care costs and risks. The disproportionate utilization of acute care setting resources is primarily attributed to irregular healthcare access; however, it is more significantly influenced by inconsistencies in care continuity during admissions to inpatient services or between inpatient hospital encounters [1-3]. In this study, we aim to understand nationwide data and how various factors influence hospital efficacy by assessing nationwide hospital length of stay (LOS). We propose innovative, data-driven interventions to improve the static nature of health information exchange (HIE) and electronic health records (EHR) systems. Our goal is to enhance access to healthcare resources, ensure continuity of care, and ultimately improve patient outcomes while avoiding the undesired consequences of data transfers. While HIE between hospitals and insurance companies aims to enhance care coordination and operational efficiency, it can also lead to several adverse outcomes [4]. Privacy breaches are a significant concern; unauthorized access to sensitive patient data can result in identity theft and violations of regulations like the Health Insurance Portability and Accountability Act (HIPAA). For instance, a ransomware attack on UnitedHealth Group’s Change Healthcare unit in February 2024 potentially compromised the health information of one-third of Americans, highlighting the vulnerability of such exchanges to cyber threats [5]. Interoperability challenges also pose risks, as inconsistent data formats and systems can lead to incomplete or inaccurate information exchange, adversely affecting patient care and claims processing. It's important to highlight that insurance companies' responsible use of shared data can lead to more personalized and fair premium adjustments. When insurance providers utilize health trends to offer tailored coverage options, it can enhance patient trust and encourage proactive health management.

Patients may feel more empowered and confident in their insurance choices with transparent communication about how data are used. Administrative burdens may increase due to the need for hospitals to comply with varying insurer data requirements, potentially leading to claim denials and financial strain. To mitigate these issues, robust cybersecurity measures, standardized data protocols, and transparent data usage policies are essential to protect patient information and maintain trust in the healthcare system [6]. We observe that LOS may reflect disparities in care quality or the availability of timely interventions, unduly affecting vulnerable populations needing assistance with discharge planning, follow-up care, or social support. Recently, the LOS has shifted control away from the patient, placing it in the hands of unpredictable factors that may not relate to the patient’s autonomy or individual characteristics and social determinants of health (SDOH). Marginalized groups may experience longer LOS due to delays in receiving needed services or managing co-morbidities. At the same time, shorter-than-necessary stays can lead to inadequate care and higher readmission rates [7]. Addressing these disparities by optimizing LOS based on patient needs rather than resource constraints is essential for equitable healthcare, as it ensures that all patients, regardless of background, receive appropriate and timely care that supports recovery and long-term health [5-7].

Prolonged hospital stays can reveal underlying throughput issues that are not necessarily related to a patient's condition, highlighting deficiencies in the care delivery or cost coverage processes. Delayed laboratory studies or diagnostic imaging often lead to patient dissatisfaction due to discomfort, uncertainty, and delays in receiving necessary care. Sometimes, patients may be admitted to acute care without a clearly defined and time-oriented treatment plan, resulting in prolonged hospital stays. Conversely, some patients remain highly engaged in their treatment during hospitalization, owing to their care teams' readiness to address care fragmentation, enhancing the quality and continuity of care. This variation emphasizes the need for a standardized approach that prioritizes nationwide patient care experiences and promotes health equity. Hospital strategies focusing on the "thrust of care" are more likely to optimize care efficiency by closely monitoring data accuracy. This approach ensures patients are admitted based on specific signs or symptoms indicating an underlying condition, with apparent medical necessity, treatment objectives, and timelines in mind. Subsequently, it minimizes delays and avoids unnecessary procedures, potentially including expedited diagnostics and suitable interventions that avoid extended observation periods, abrupt admission orders, and unclear discharge dispositions.

This is why effective LOS management is essential for balancing clinical outcomes, avoiding potentially preventable complications and PPCs, and improving patient safety and overall patient experience without compromising the quality of care provided. Numerous studies have concluded that morbidity rates are closely related to ineffective rescue measures caused by unnecessary treatments or disrupted LOS maneuvers [8,9]. Current LOS embedded in EHRs often falls short of accommodating the complexities introduced by an aging population or associated co-morbidities [10]. Traditional LOS metrics rely on historical averages or predetermined benchmarks that may not adequately reflect the unique needs and prolonged recovery times ordinary among older patients. It is reasonable to conclude that the aging population significantly influences healthcare systems globally, particularly in the United States, where an increasing number of hospitalized elderly patients presents unique challenges and preferences [11]. Older adults often experience prolonged LOS due to multiple co-morbidities and the necessity for specialized care, which leads to higher resource demands. This situation can strain hospital capacity, escalating healthcare costs and negatively impacting performance metrics. The standard LOS models might not account for the entire continuum of care, including the need for rehabilitation or time to guard against the escalating complication risk from infections or mobility-related injuries [12].

Moreover, it is important to consider patients discharged from psychiatric or surgical wards frequently require additional inpatient admissions for ongoing medical management and medication reconciliation, creating a cyclical pattern of care that presents both opportunities and risks. While successive hospital encounters can enhance care coordination and improve medication management, they also pose challenges such as increased hospitalization rates, potential stigmatization, and fragmented care. To mitigate these risks, it is imperative to implement integrated care models, establish robust follow-up services, and adopt patient-centered approaches [12-15].

While co-morbidities and pre-existing conditions impact hospitalization experiences, so do deficiencies in HIE, which can adversely affect hospital LOS by hindering the efficient sharing of patient information among healthcare providers. When HIE systems are inadequate or underutilized, clinicians may need more timely access to critical patient data, such as prior hospitalization histories, leading to delays in nationwide healthcare interoperability shortcomings. Patients are often transferred between hospital units or from and onto different hospitals to seek specialized care. Transfers frequently interrupt the continuity of care, potentially leading to information gaps and miscommunication. Patient safety and care outcomes can be compromised when patient records and treatment details are not effectively exchanged. Inter-hospital transfers, when patients move between hospitals, often experience longer LOS. This extension can result from the time required for transfer coordination, additional assessments upon arrival, and potential delays in initiating appropriate treatments. Studies such as that of Rishu et al. have shown that transferred patients may have more extended hospital stays than those admitted directly, even after adjusting for illness severity. Unlike intra-hospital transfers, moving patients to higher levels of care within the same hospital (e.g., from a general ward to the ICU) can also prolong LOS. Such transfers may indicate clinical deterioration, necessitating more intensive and extended care. Research suggests that patients requiring intrahospital transfers to higher care levels often have increased LOS and higher mortality rates [16].

A systematic review published in the Journal of General Internal Medicine in 2022 examined the effects of HIEs in adult inpatient settings. The review found that, while some studies reported benefits of HIEs, such as reduced readmission rates and mortality, the overall evidence was mixed, and many studies showed no significant impact on LOS. The authors noted that the variability in outcomes could be attributed to differences in HIE implementation and utilization across institutions [17]. Conversely, a study analyzing data from Massachusetts emergency departments found that HIE adoption was associated with an 11.1% reduction in LOS, increasing to 16.5% for patients with previous visits to HIE-participating hospitals. This suggests effective HIE implementation can enhance information coordination, reducing LOS [18].

Previous statistical briefs, such as "Potentially Preventable Hospitalizations among Medicare-Medicaid Dual Eligibles" from September 2008, highlight the significant impact of a patient's insurance on hospitalization and recovery duration. Furthermore, understanding the relationship between Medicare and Medicaid enrollment is vital in healthcare delivery, particularly for dual-eligible beneficiaries who qualify for both programs [19]. These patients frequently present complex medical needs that lead to prolonged hospital stays due to severe conditions and restricted access to primary and preventive care services. While Medicare primarily covers acute care hospital services, Medicaid may supplement this coverage to address additional costs not reimbursed by Medicare. This interplay can lead to more extended hospital stays as patients may require more comprehensive care that Medicare does not fully cover. Understanding these dynamics is crucial for improving patient outcomes and optimizing healthcare delivery systems, mainly through aligning the distinct care models of Medicare and Medicaid. Initiatives such as the Medicare-Medicaid Financial Alignment Initiatives and Dual-Eligible Special Needs Plans (D-SNPs) aim to enhance integration and coordination between these two programs, ultimately improving care outcomes and reducing costs.

In response, accountable care organizations (ACOs) evolved to represent a collaborative approach among healthcare providers, including physicians, hospitals, and various allied entities, designed to deliver coordinated, high-quality care to Medicare beneficiaries. These organizations play a crucial role in managing patient care by implementing strategic measures to optimize healthcare delivery, enhance patient outcomes, and minimize healthcare costs. ACOs strive to reduce the LOS for patients in healthcare facilities by ensuring that patients receive appropriate care at the right time. This collaborative framework emphasizes the avoidance of unnecessary service duplication and preventing medical errors, thereby fostering efficiency and enhancing the overall quality of care. Through these efforts, ACOs improve patient experiences and contribute significantly to the sustainability of healthcare systems [20].

ACOs are typically part of a value-based care model, which ties provider reimbursements to the quality of care rather than the quantity of services. When an ACO successfully meets specific quality benchmarks and reduces healthcare spending for its patient population, it can share in the savings with Medicare. However, if the ACO does not meet the targets or incurs higher costs, it may face financial penalties. ACOs prioritize care coordination among healthcare providers to mitigate treatment delays and facilitate timely discharges. This involves implementing effective discharge planning processes that commence at the point of admission and offering comprehensive education to patients regarding their conditions and post-discharge care.

Additionally, ACOs emphasize preventive care and the management of chronic conditions to minimize the risk of hospitalization. They conduct utilization reviews to evaluate the necessity of continued hospitalization and work collaboratively with post-acute care providers to ensure adequate care transition following discharge. By leveraging data analytics, ACOs can track LOS metrics, identify trends, and pinpoint areas for improvement. The multidisciplinary teams within ACOs cater to the diverse needs of patients, promoting quicker and more efficient care delivery. These concerted efforts aim to enhance patient outcomes, improve patient experience, and reduce the financial burdens of prolonged hospital stays. The development of ACOs has prompted a fundamental shift in the healthcare landscape. However, many hospitals need help joining these alliances. This reluctance often stems from the substantial cultural and operational readiness required for effective participation. Embracing ACOs necessitates a commitment to value-based care, which involves transforming existing practices, reorganizing workflows, and fostering a collaborative culture focused on patient outcomes. Consequently, hospitals must assess their readiness for these significant modifications to align with the principles of value-driven healthcare.

Thus, the intersections of vertical and horizontal strategies nationwide and within the rapidly evolving hospital operations must be strategically aligned to prioritize patients' well-being and health outcomes. Understanding that each patient's journey through hospitalization is inherently unique and influenced by many interrelated factors highlights the complexity surrounding the LOS in acute care settings. These factors shape hospital operational dynamics and profoundly affect disease severity and quality outcomes. Therefore, it is imperative to develop robust predictive models that empower healthcare providers to make evidence-based decisions regarding anticipated LOS. Such models can foster meaningful connections by integrating insights about the interplay between a patient's clinical status and their social determinants of health, thereby facilitating a more personalized and comprehensive approach to patient care.

LOS and medical necessity

The interplay between LOS in healthcare facilities and medical necessity is critical in delivering effective patient care. Medical necessity pertains to services deemed appropriate for diagnosing or treating a medical condition, establishing an essential foundation for determining LOS. When a patient's stay is classified as medically necessary, it aligns with an optimal LOS, ensuring that patients receive adequate care until they are sufficiently stable for discharge. Conversely, when a patient's condition necessitates ongoing treatment or monitoring - common in cases involving complex surgeries or severe illnesses - LOS may need to be prolonged. This concept is mainly retrospective and depends on data mining techniques, which can be vague due to the disconnect between documentation and reality. In some cases, documentation may inadequately reflect the patient's clinical condition, resulting in denials for "lack of medical necessity." This often occurs because EHR templates do not adequately capture the individual nuances of each patient in a timely or at scale [21].

A pivotal entity in this dynamic is insurance companies, which assess the medical necessity of treatments to establish reimbursement eligibility. Lengths of stay exceeding the established medical necessity benchmarks can lead to denied coverage for the additional days. It is noted that some hospitals are using different benchmarks to access medical necessities that may differ from their corresponding insurance. Under Title 42, §482.30, hospitals are permitted by CMS to select their utilization review tools. Thus, hospitals may incur fiscal penalties due to elevated LOS for specific conditions, compelling them to emphasize the justification of stays focused on medical necessity without having sufficient tools to predict outcomes based on patient characteristics prospectively. This complex relationship impacts the quality of care, as providers need to balance delivering necessary care with optimizing resource use. The financial ramifications for healthcare facilities and patients regarding extended stays that lack medical necessity can be profound; insurers are likely to reject claims for days perceived as unwarranted, resulting in financial setbacks and potential out-of-pocket expenses for patients. The current clinical decision models, which aim to explore pivotal determinants of LOS and are used by hospitals, often fall short in addressing the increasingly nuanced nature of patient needs, primarily due to restricted data inputs. Many conventional tools concentrate narrowly on clinical data, frequently overlooking vital elements, such as social determinants of health, mental health status, socioeconomic factors, and patient preferences - all crucial factors significantly impacting LOS.

Managed care guidelines and LOS visibility

InterQual and Milliman Clinical Guidelines (MCG) are critical frameworks most hospitals utilize in the healthcare sector to assess medical necessity across various levels of care. InterQual provides criteria for evaluating patients' needs based on clinical evidence to optimize admission decisions for inpatient, outpatient, and rehabilitation services. By emphasizing a patient's current condition, expected outcomes, and related risks, InterQual facilitates a standardized approach to care management and documentation. This consistency is vital for optimizing reimbursement processes in healthcare settings. However, despite its structured approach, InterQual may need help adapting to individual patients' unique circumstances. The rigid application of guidelines can overlook essential factors such as social determinants of health, multi-morbidity complexities, and patient preferences. This one-size-fits-all methodology risks leading to inappropriate care decisions if healthcare providers rely too heavily on standardized criteria without considering the diverse needs of each patient. 

Similarly, MCG plays an instrumental role in determining medical necessity through evidence-based guidelines designed to assist healthcare providers in making well-informed treatment decisions. By guiding care utilization and ensuring alignment with best practices, MCG enhances the quality of care and facilitates smoother reimbursement navigation. Nonetheless, MCG may present challenges due to its inherent rigidity. While the structured guidelines promote adherence to established evidence-based practices, they can inhibit clinical judgment, particularly in cases involving atypical patient presentations or complex needs. In such situations, the inability to fully integrate personalized care considerations can lead to suboptimal patient outcomes. A recent US Department of Health and Human Services Office of Inspector General (OIG) report found that 13% of commercial Medicare Advantage care denials should have been covered under Medicare. Additionally, about 18% of legitimate Medicare Advantage claims were denied despite meeting Medicare coverage rules. Such denials occur because commercial insurers frequently follow established clinical guidelines, such as MCG or InterQual, and make “proprietary” modifications to them to adjudicate prior authorization requests and claims [22].

InterQual and MCG offer essential frameworks to support medical necessity determinations; however, they come with limitations that healthcare providers must navigate. InterQual's potential oversights regarding unique patient circumstances contrast with MCG's restrictive nature, which may undermine clinical flexibility. Physicians must balance guideline adherence and individualized assessment for optimal patient care, utilizing additional tools and decision-making frameworks that enable a more specified approach to clinical practice. This balance is imperative to address patient needs adequately while aligning with evidence-based care standards.

Value-based care and influence on LOS

The value-based care (VBC) model emerged as a pivotal response to significant findings in landmark reports, notably, the Institute of Medicine's "To Err is Human" (1999) and "Crossing the Quality Chasm" (2001) [1,23]. These documents illuminated critical deficiencies in patient safety, quality of care, and operational efficiency within healthcare systems. Consequently, the VBC philosophy shifted the emphasis from the quantity of services rendered to the quality of care delivered to patients. This transformative approach aims to enhance patient outcomes while controlling healthcare costs, fostering a paradigm shift from volume-driven practices to value-oriented care. Implementing VBC carries profound implications for concepts such as medical necessity and LOS in various healthcare settings, necessitating a reevaluation of traditional metrics and strategies in patient management. One of the primary impacts of value-based care on medical necessity is the increased emphasis on evidence-based practices and patient-centered care. Under VBC, healthcare providers are incentivized to ensure that treatments and interventions are necessary to improve patient health outcomes. This focus encourages providers to rigorously use clinical guidelines and decision-support tools to assess medical necessity. As a result, unnecessary admissions and procedures may be reduced, leading to more appropriate care levels that align with patients' actual needs.

Regarding LOS, value-based care promotes efficient care delivery, which can lead to shorter hospital stays without compromising patient safety or outcomes. By prioritizing timely interventions and effective care coordination, providers can facilitate quicker discharges for patients who no longer require inpatient care. This efficiency benefits patients by reducing their time in the hospital, helps healthcare organizations manage resources more effectively, and reduces costs associated with prolonged stays. Additionally, shorter lengths of stay can positively impact hospital readmission rates, as patients are often discharged when they are stable and ready for follow-up care. However, the transition to value-based care also presents challenges. Providers may face pressure to reduce lengths of stay, which, if not managed carefully, could lead to premature discharges. Ensuring patients receive appropriate follow-up care and support after discharge is crucial to avoid adverse outcomes, such as readmissions or complications.

Furthermore, the shift to VBC requires robust data analytics and care coordination efforts to monitor patient outcomes effectively and adjust care plans. In summary, value-based care significantly influences medical necessity and LOS by promoting evidence-based practices and efficient care delivery. While it encourages appropriate treatment decisions and shorter hospital stays, careful management is essential to ensure that patient safety and quality of care remain priorities throughout the transition to this model.

Geometric mean LOS (GMLOS)

The GMLOS is a critical statistical measure in the healthcare sector, optimizing the assessment of patients’ average LOS within specific diagnosis-related groups (DRGs) or treatment categories. This method attempts to improve the arithmetic mean, which can be distorted by outliers, thereby offering a more representative metric for central tendency, particularly in datasets characterized by substantial variability. In contemporary value-based care models and reimbursement systems, GMLOS is increasingly leveraged to benchmark institutional performance against the expected LOS for comparable patient populations. A systematic approach is employed to calculate GMLOS. Initially, data about the LOS for all patients within a given DRG or treatment category are amassed over a predetermined timeframe. Each documented LOS transforms a ratio through division by the geometric mean of the dataset, which serves to normalize the data. The subsequent step involves aggregating these ratios via multiplication and extracting the nth root of the resultant product, where n denotes the total patient count in the dataset. This method yields the GMLOS, presenting a more reliable average than the arithmetic mean.

Our analysis indicates that using GMLOS as a benchmarking tool can lead to the mismanagement of outliers, such as same-day discharges and specific hospital populations with lengths of stay that exceed set outlier thresholds. Using local data sourced from Arrowhead Regional Medical Center (ARMC) spanning February 2022 to December 2024, we aim to juxtapose these localized data with nationwide average LOS figures, providing a nuanced understanding of the "thrust of care" and its implications for LOS. As a pivotal community-based organization in San Bernardino County, California, ARMC is positioned to furnish integrated acute hospital care services. This investigation is oriented towards synchronizing healthcare facilities’ quality improvement initiatives and patient safety objectives with national guidelines. By utilizing nationwide data from the Centers for Medicare & Medicaid Services (CMS), we identified critical trends and factors contributing to the misalignment of LOS across hospitals. Our findings reveal that several systemic issues, including the lack of multidisciplinary rounds, delays in care coordination, and insufficient safe discharge pathways significantly influence this misalignment.

Patients whose GMLOS exceeds the 150th percentile relative to their actual LOS are categorized among peers with analogous DRG and average LOS (ALOS). This classification not only distorts the calculations underlying severity of illness (SOI) and risk of mortality (ROM) assessments but also needs to accurately represent the projected LOS for diverse patient cohorts. We observed that the ALOS frequently surpasses GMLOS, insinuating that numerous patients experience prolonged stays, further distorting the average.

The relationship between unconscious machine learning (ML) algorithms and medical decision-making has been examined extensively in various studies. For instance, MacIntyre et al. explored "Ethical considerations for the use of artificial intelligence in medical decision-making capacity assessments," highlighting that, while ML can standardize patient assessment tools, it may fall short in addressing emerging concerns regarding patient autonomy [24]. This research underscores the need to consider ethical implications when integrating AI in clinical settings carefully. However, it was found that heavy reliance on GMLOS for hospitals' performance evaluation can result in penalizing departments with patients requiring longer LOS, such as in palliative care, geriatrics, or trauma. This creates resistance among staff, as strict adherence to GMLOS may be perceived as prioritizing finances over patient care [25]. Our investigation was motivated by the necessity to gain a deeper understanding of co-morbidities, patient-centered care, medical decision-making, and social determinants of health. We aim to explore these complexities by applying advanced ML tools better suited to address the dynamic nature of evolving medical situations. We strive to improve care and outcomes for diverse populations by examining these factors.

Goals of this investigation

We aim to proactively identify information gaps and propose new insights for better data precision and synchronization protocols. Our analysis of nationwide hospital LOS data demonstrates data-driven interventions tailored to patients’ needs, aiming to improve the hospital experience and reduce care fragmentation. Given that the LOS in healthcare settings is a multifaceted metric influenced by various factors, including patient demographics, clinical presentations, the complexity of treatments administered, the occurrence of complications, and the processes involved in discharge planning. However, it is critical to recognize that these significant factors do not inherently enhance patient empowerment throughout their healthcare recovery journey. Empowerment in this context is mainly contingent upon patients' quality of communication regarding their diagnoses, treatment modalities, and anticipated outcomes. These factors are essential for enabling patients to make informed decisions regarding their care.

Relying on this understanding, the present study focuses on patients' needs and experiences during inpatient hospitalization. This shift is imperative for fostering an environment where patients feel engaged and informed. Current EHR systems do not allow patients to provide their essential input, nor do they offer providers concurrent gradient to leverage patient experiences. Thus, we comprehensively evaluate nationwide data to accomplish this objective, facilitating a nuanced comparison of observed versus expected LOS across diverse healthcare settings. Additionally, highlight our findings from a local perspective by studying the LOS data at ARMC and associated DRGs. This methodological approach underscores the necessity of utilizing concurrent tools that can scale from a localized perspective to a broader national framework. Moreover, the study highlights the critical importance of continuous quality assessment regarding hospital LOS. This entails the measurement of LOS and the development of innovative tools capable of predicting, analyzing, intervening, and prompting concurrent action based on the insights gained from data analysis. By integrating these components, the study aspires to encapsulate its core objectives: to enhance patient empowerment through improved communication, to refine the assessment of LOS via innovative approaches, and to develop predictive tools that can inform clinical practice and policy. Ultimately, this research contributes to a more patient-centered approach to managing inpatient care, improving patient outcomes and satisfaction.

## Materials and methods

This study uses statistical methodologies to thoroughly examine the relationship between hospital LOS and critical health variables, in line with our ARMC protocol 22-40, titled "Impact of Timely Production of Health Information on Patient Safety and Hospital Quality Outcomes at ARMC." We rely on nationwide data from the CMS, accessed in December 2024, which includes segmented inpatient hospital data by region, period, demographics, LOS, insurance coverage, mortality rates, and other relevant factors [26]. This comprehensive framework enhances our understanding of how these elements interact and influence patient care outcomes in a hospital setting. Additionally, we incorporate local data from patients who stayed longer than 18 days at ARMC between February 2022 and December 2024, along with information on associated disease categories. Our goal is to highlight the impact of each variable on LOS by assigning a score that reflects this influence while minimizing the noise introduced by interactions among other variables. By visualizing LOS concerning other factors and assigning propensity scores, we present an effective method for understanding and predicting patient outcomes tailored to each patient's unique characteristics. This approach also offers a means of standardization that can be applied to hospital systems or populations in real time. Inclusion criteria from the ARMC data search include patients with complete data on demographics, LOS, insurance coverage, and mortality rates, and their cases should align with specific disease categories relevant to the study. We also utilized nationwide data from CMS to ensure a comprehensive analysis. Conversely, we excluded patients with 18 days or less LOS and individuals with incomplete data on key variables. Additionally, we excluded patients with transient conditions that do not reflect typical hospital stays and any data outside the specified timeframe. This approach aims to focus on a relevant patient population, minimizing confounding factors and enhancing the validity of our findings regarding the impact of timely health information production on patient safety and hospital quality outcomes.

We conducted the nationwide analysis in two stages. The first stage utilizes descriptive statistics to identify general trends across different CMS datasets, while the second stage employs more detailed inferential methods, including adding propensity scores. The descriptive analysis offers insights into averages, variations in health status, and the LOS. Synchronously with thorough analysis, propensity score matching (PSM) is applied to account for differences in patient health information in the primary data, allowing for a more precise assessment of the impact of health variables on LOS. Additionally, we performed a regression analysis to investigate the relationship between specific health conditions and LOS. PSM was performed using the nearest neighbor algorithm with a caliper width of 0.1 to ensure precise matching. Examples of key variables for matching included age, LOS, and morbidity. Balance diagnostics were evaluated using standardized mean differences (SMD), with all covariates meeting the predefined threshold of <0.1 to confirm adequate balance. Regression analyses were subsequently conducted in R (version 4.3; R Development Core Team, Vienna, Austria) utilizing the glm function, with adjustments for SDOH variables, such as income and education levels to account for confounding factors. The visualization of results was enhanced through heat maps generated in Python using the seaborn library, employing a normalized color scale to represent correlation coefficients accurately and highlight relational patterns. This multi-pronged statistical approach enhances the validity of the results and allows for identifying potential confounding factors that may influence LOS. Furthermore, integrating these datasets facilitates the triangulation of data approaches, enriching the analysis and providing a more focused understanding of the factors affecting hospital LOS. By balancing the broad patterns observed in the national data with the focused insights derived from the PSM, the study aims to contribute to the existing literature on hospital utilization and patient care, offering recommendations for policy and practice informed by national trends and ideal data realities.

The Medicare Inpatient Hospital reports offer extensive raw data concerning patients under Medicare, focusing on various aspects of hospital utilization of Medicare resources, payments, and cost-sharing for original Medicare beneficiaries from 2011 to 2021. These reports are essential for analyzing variations based on entitlement types and monitoring yearly trends. They provide insights into demographic information, including Medicare-Medicaid enrollment status, geographic location, and the types of hospitals involved. However, while these reports are rich in information, they must provide the concise data necessary for daily hospital management, bed allocation, or HIE functions. Consequently, our study emphasizes the experience of Medicare Inpatient Prospective Payment System (IPPS) long- and short-stay hospitals, examining patterns related to utilization, payments, and cost-sharing mechanisms differentiated by entitlement type and demographic characteristics. Geographic considerations further enrich the analysis, allowing a comprehensive understanding of how hospital location, bed size, medical school affiliation, and hospital type interact. Additionally, the reports encompass insights into special-category hospitals, detailing their utilization trends, program payment frameworks, and cost-sharing arrangements specifically for original Medicare beneficiaries. This thorough approach renders these reports an invaluable resource for researchers and policymakers seeking a deeper understanding of the complexities of the Medicare inpatient landscape.

Aligning care delivery with value-based care principals

Our analysis commenced with a detailed examination of the relationship between patient age, LOS, and discharge outcomes. We utilized Statistical Product and Service Solutions (SPSS, version 27; IBM SPSS Statistics for Windows, Armonk, NY) to conduct comprehensive data analysis to identify correlations among critical variables, mainly focusing on patient age and the total duration of care. To assess the relationships between hospital stay length and discharge mortality across different age brackets, we employed Pearson correlation coefficient and two-tailed significance testing to capture the nuances of these associations. Descriptive statistics played a crucial role in our analysis, offering a foundational understanding of patient demographics and general trends in hospital stays. This preliminary exploration laid the groundwork for a more intricate, correlation-focused data investigation. The primary objective was to pinpoint significant correlations that might illuminate patterns in patient outcomes, emphasizing how age and length of hospital care could impact mortality scores. Leveraging the robust functionalities of SPSS allowed us to effectively manage and interpret the data, revealing meaningful associations that underscore the implications of age-specific hospital stays on patient outcomes. Our findings, supported by descriptive statistics, are illustrated through the analysis of CMS data from 2011 to 2021, as depicted in Table 1.

**Table 1:** Correlations among nationwide length of stay and mortality among different age groups * Correlation is significant at the 0.05 level (2-tailed); ** Correlation is significant at the 0.01 level (2-tailed).

Correlations				
Age			Total Days of Care	Discharged Dead
Under 18 Years	Total Days of Care	Pearson Correlation	1	-.991^**^
		Sig. (2-tailed)		0.009
		N	8	4
	Discharged Dead	Pearson Correlation	-.991^**^	1
		Sig. (2-tailed)	0.009	
		N	4	4
18-24 Years	Total Days of Care	Pearson Correlation	1	0.972
		Sig. (2-tailed)		0.152
		N	8	3
	Discharged Dead	Pearson Correlation	0.972	1
		Sig. (2-tailed)	0.152	
		N	3	3
25-34 Years	Total Days of Care	Pearson Correlation	1	0.565
		Sig. (2-tailed)		0.056
		N	12	12
	Discharged Dead	Pearson Correlation	0.565	1
		Sig. (2-tailed)	0.056	
		N	12	12
35-44 Years	Total Days of Care	Pearson Correlation	1	0.183
		Sig. (2-tailed)		0.57
		N	12	12
	Discharged Dead	Pearson Correlation	0.183	1
		Sig. (2-tailed)	0.57	
		N	12	12
45-54 Years	Total Days of Care	Pearson Correlation	1	.655^*^
		Sig. (2-tailed)		0.021
		N	12	12
	Discharged Dead	Pearson Correlation	.655^*^	1
		Sig. (2-tailed)	0.021	
		N	12	12
55-64 Years	Total Days of Care	Pearson Correlation	1	0.408
		Sig. (2-tailed)		0.188
		N	12	12
	Discharged Dead	Pearson Correlation	0.408	1
		Sig. (2-tailed)	0.188	
		N	12	12
65-74 Years	Total Days of Care	Pearson Correlation	1	-0.004
		Sig. (2-tailed)		0.989
		N	12	12
	Discharged Dead	Pearson Correlation	-0.004	1
		Sig. (2-tailed)	0.989	
		N	12	12
75-84 Years	Total Days of Care	Pearson Correlation	1	0.045
		Sig. (2-tailed)		0.889
		N	12	12
	Discharged Dead	Pearson Correlation	0.045	1
		Sig. (2-tailed)	0.889	
		N	12	12
85-94 Years	Total Days of Care	Pearson Correlation	1	0.481
		Sig. (2-tailed)		0.114
		N	12	12
	Discharged Dead	Pearson Correlation	0.481	1
		Sig. (2-tailed)	0.114	
		N	12	12
95 Years and Over	Total Days of Care	Pearson Correlation	1	-0.237
		Sig. (2-tailed)		0.651
		N	6	6
	Discharged Dead	Pearson Correlation	-0.237	1
		Sig. (2-tailed)	0.651	
		N	6	6
95 Years or Over	Total Days of Care	Pearson Correlation	1	-0.344
		Sig. (2-tailed)		0.505
		N	6	6
	Discharged Dead	Pearson Correlation	-0.344	1
		Sig. (2-tailed)	0.505	
		N	6	6

This comprehensive approach facilitates a deeper understanding of the interplay between patient age, hospital stay duration, and subsequent discharge outcomes. Investigating the relationship between hospital encounter length and mortality rates reveals remarkable insights across different age demographics. Our analysis indicates a notable disparity in outcomes based on age when considering the duration of hospitalization.

**Figure 1:**
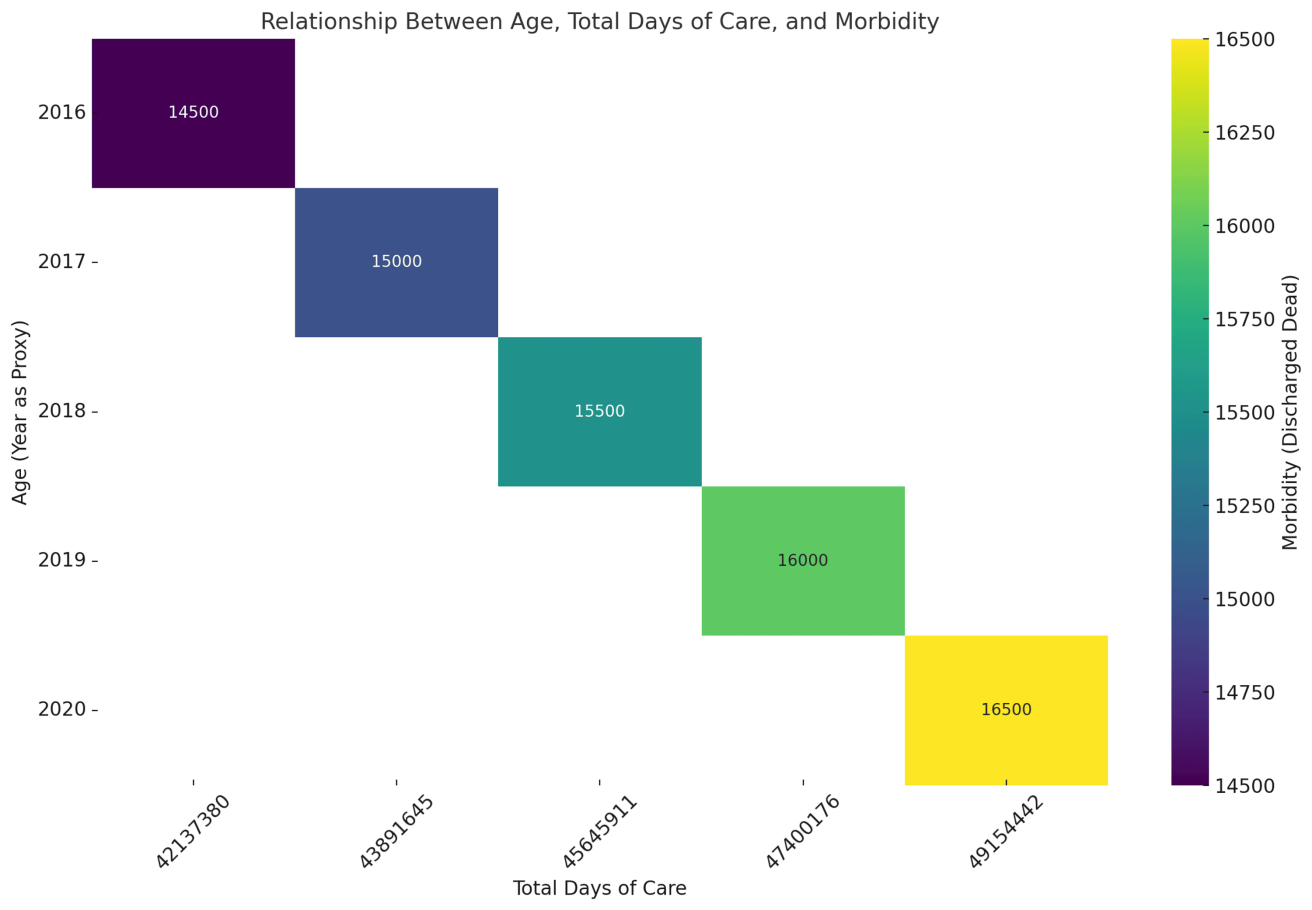
Age, length of stay, and morbidity trends nationwide (2016-2020) The graph showcases the relationship between age (using years as a proxy), total days of care, and morbidity (measured as discharged dead) from 2016 to 2020. Over the years, both total days of care and morbidity have shown a steady increase. The x-axis represents the total days of care, which rises annually, and the color gradient—from dark purple to bright yellow—illustrates the corresponding increase in morbidity, ranging from 14,500 in 2016 to 16,500 in 2020. Each block represents a specific year, with the values labeled for clarity. The trend suggests a growing healthcare burden over time, as more care results in higher morbidity. This visualization emphasizes the opportunities for improvement in healthcare systems as we address rising care demands and strive for better health outcomes.

For patients under 18, we observe a robust negative correlation, with a Pearson correlation coefficient of -0.991 (p = 0.009). This significant negative relationship suggests that, as the total days of care increase, the likelihood of being discharged deceased diminishes. Such findings imply that extended hospital care may be particularly effective for younger patients, highlighting the potential benefits of proactive medical intervention. Contrastingly, in the 18-24 age demographic, the correlation shifts to a positive, albeit non-significant, relationship at 0.972 (p = 0.152). While the lack of statistical significance warrants caution in interpretation, this correlation may hint at a trend where prolonged hospital stays coincide with a higher rate of discharge deaths. This could reflect the severity of health conditions necessitating extended care among this age group. Moving to the 25-34 age group, we find a moderate positive correlation of 0.565 (p = 0.056). Although this correlation is below statistical significance, it suggests a potential trend, indicating that increased LOS correlates with a higher likelihood of discharge mortality. This may indicate underlying complex health issues in patients requiring more extended hospitalization. The correlation strengthens in the 45-54 age bracket, yielding a statistically significant Pearson coefficient of 0.655 (p = 0.021). This positive relationship points to the association between extended care days and an increased likelihood of discharge death within this age group, suggesting that patients in critical health conditions may require prolonged hospitalization. For the 55-64 cohort, the correlation decreases to a weak positive value of 0.408 (p = 0.188). While this indicates some degree of association, it does not reach statistical significance, implying that more extended hospital stays do not strongly predict higher discharge mortality within this demographic. The older age groups (65-74, 75-84, and 95+) exhibit correlations that suggest minimal meaningful relationships between total days of care and discharge deaths (-0.004, p = 0.989; 0.045, p = 0.889; and -0.237, p = 0.651, respectively). These findings imply that, for these elderly patients, the LOS may not significantly influence the likelihood of mortality, potentially due to higher baseline risks inherent to advanced age. Lastly, the 85-94 age group presents a moderate positive correlation of 0.481 (p = 0.114), although it remains statistically insignificant. This observation may indicate a slight trend suggesting that more extended hospitalizations coincide with increased discharge mortality, likely reflecting the impact of more complex health conditions among these patients. Ultimately, our analysis underscores the complexity of relationships between length of hospital stay and mortality rates across various age brackets, suggesting that age-specific factors play a crucial role in understanding hospital outcomes, as shown in Figure 1.

The abovementioned approach carries significant implications for the future trajectory of healthcare, particularly in predictive analytics and personalized medicine. These covariates establish a foundation for developing predictive models that assess real-time risks during hospital admissions, which are currently lacking, by uncovering the correlations between patient age, LOS, and mortality rates. Such models could facilitate tailored care planning that aligns with individual patient profiles, ultimately enhancing resource allocation and clinical decision-making processes. Integrating these insights into AI-powered systems within EHRs has the potential to enable continuous risk assessments and bolster clinical decision support. Such advancements promise to improve patient outcomes and operational efficiency within healthcare settings. The implications of studying themes emerging from hospital dynamics also extend to health policy and hospital operations. By clarifying the connections between age, duration of hospitalization, and mortality, the study can guide quality improvement initiatives, particularly age-specific interventions aimed at reducing adverse outcomes and refining discharge processes. These insights can enhance existing metrics, including CMS star ratings and leapfrog safety scores, by weaving in more sophisticated predictors of mortality. Hospitals could utilize these data to streamline resource allocation, predict staffing requirements, and implement targeted interventions for vulnerable populations, aligning care delivery practices with value-based care principles. Finally, this research addresses broader issues surrounding healthcare equity and population health management. The analysis can inform public health interventions designed to mitigate risks for underserved populations by illuminating patterns and disparities in care outcomes across various age demographics. Additionally, it supports longitudinal studies evaluating the effects of shifting care models and demographic trends, including aging populations, on healthcare outcomes over time. In advancing healthcare technologies, these findings could serve as a foundation for training ML models, paving the way for creating sophisticated tools capable of predicting outcomes effectively and enhancing the overall quality of patient care.

Financial metrics and impact on future trajectories 

**Figure 2:**
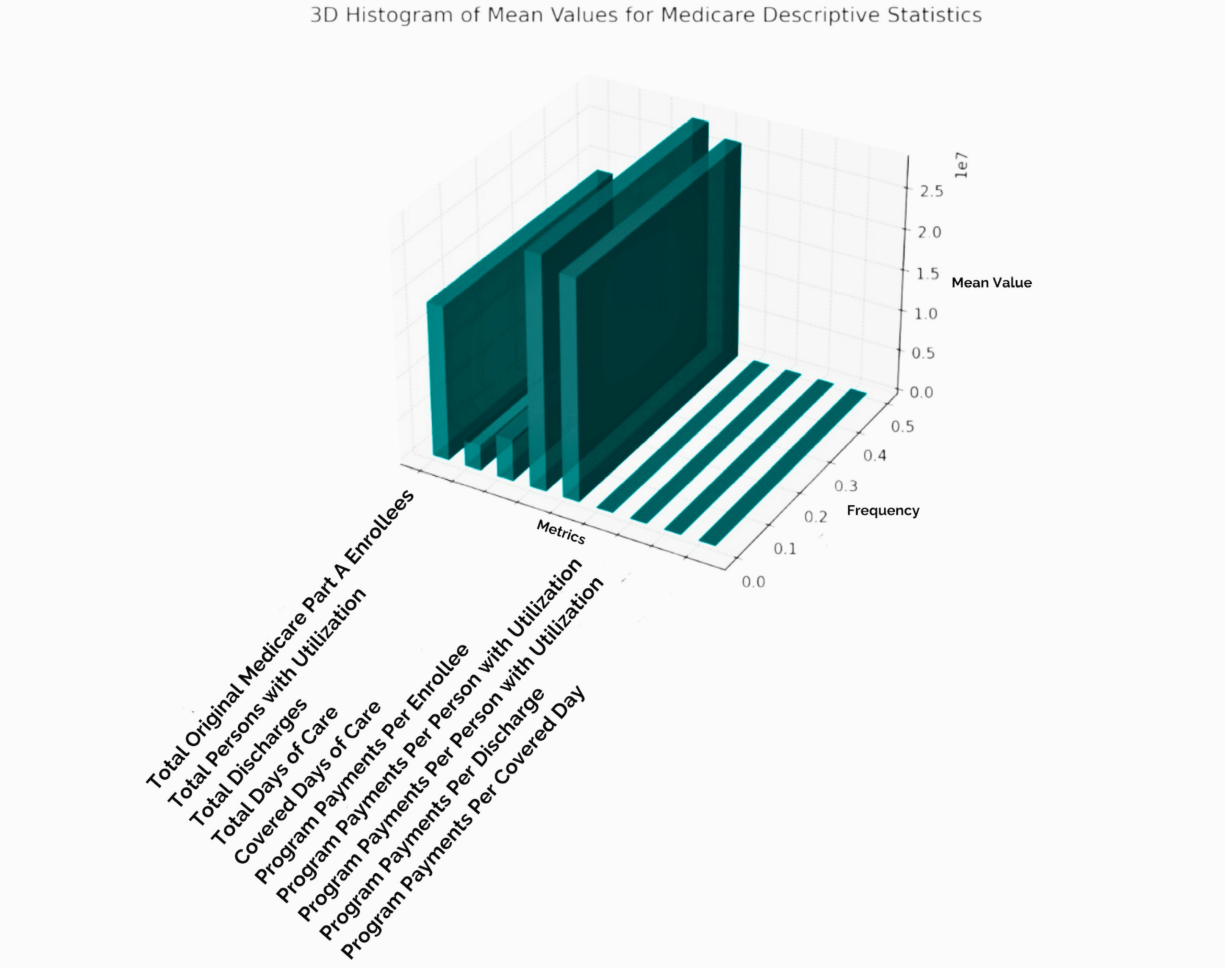
3D histogram of the mean values for Medicare descriptive statistics (2011-2021) The 3D histogram visualizes the mean values of various Medicare metrics, with each bar representing a different metric (e.g., “Total Medicare Part A Enrollees,” “Total Days of Care,” etc.). Each bar's height corresponds to that metric's average value, allowing for quick comparison across metrics. Taller bars, like those for “Total Medicare Part A Enrollees” and “Total Days of Care,” indicate higher mean values. In comparison, shorter bars represent metrics with lower averages, such as “Program Payments Per Covered Day.” This chart effectively highlights the relative scale of each metric, providing insight into which areas have larger or smaller average values.

The evaluation of financial metrics associated with the LOS for Medicare-covered patients needs to be explored more in the literature. This oversight significantly affects hospital resource utilization and can lead to miscalculated outcomes when employing traditional models, particularly from the perspective of insurance benefits. The implications of this issue are further illustrated in the descriptive statistical analysis presented in Figure 2, underscoring the necessity for a more comprehensive understanding of LOS dynamics and their financial ramifications. Such analysis is critical to optimizing hospital operations and improving patient care outcomes. The investigation focuses on key metrics such as total days of care, program payments, and coinsurance structures, which are vital for comprehending the dynamics of LOSs and their broader patient-centered financial prospects. This viewpoint of data interpretation provides valuable insights for patients and healthcare institutions, contributing to a better understanding of the interplay between economic factors and patient care outcomes.

Starting with the number of Medicare Part A enrollees, the data reveal a significant range, with the smallest group totaling just over 4.4 million and the largest surpassing 32 million, resulting in an average enrollee count of around 18.7 million. This variability hints at substantial shifts in enrolment that could be due to differences in population demographics, policy changes, or geographic factors across different regions.

Utilization metrics show similar variability, representing the number of people receiving care. The dataset indicates that anywhere from approximately 660,000 to 5.7 million people accessed Medicare Part A services, with a mean utilization figure of around 3.1 million. The "Total Discharges" metric, which counts hospital discharges, ranges broadly from a minimum of about 1.1 million to a high of 9.5 million. This range underscores the diversity in healthcare needs among enrollees and potential differences in hospital use patterns across the country. Additionally, the average discharges per 1,000 Medicare enrolees and 1,000 persons with utilization show steady rates of service engagement, with the former averaging about 294 discharges per 1,000 enrolees and the latter at approximately 1,718 discharges per 1,000 users. These statistics show the frequency of hospitalizations relative to the enrolled and actively treated populations.

When examining total days of care, the dataset further emphasizes the scope of hospital utilization. With total days ranging from 7.1 million to over 55 million, there is a clear reflection of the extensive time spent in care among enrollees. On average, each 1,000 Medicare enrollees required around 1,748 days of care, with this figure rising among individuals actively using Medicare services. Notably, the average daily discharge is around 5.8 days, suggesting a typical hospital stay is close to a week. Covered days of care, which represent the subset of days Medicare paid for, follow similar trends in magnitude and variation.

The financial component reveals substantial program payments to cover Medicare Part A enrolees' healthcare expenses. The total program payments range from $18.7 billion to over $112 billion, with an average expenditure close to $61 billion. Payments per enrolee and per person using Medicare vary as well, with each enrolee costing Medicare roughly $3,586 on average and each user about $21,106. These figures illustrate the financial scale of Medicare's role in supporting hospital care and the potentially high cost per patient. Additionally, program payments per discharge (around $12,290) and per covered day (around $2,293) provide insight into the cost dynamics of hospital services.

The data also cover coinsurance, representing out-of-pocket costs that some patients are responsible for. The total coinsurance days range from approximately 247,000 to 1.25 million, while the average coinsurance days per person are about 14.8. Coinsurance payments vary widely, with individual costs averaging around $9,296, underscoring the financial burden that certain enrollees may face, depending on the care they require. Further metrics include deductible payments, lifetime reserve days, and the number of patients discharged deceased, each offering additional insight into Medicare Part A's complexity and the diverse healthcare needs of its population. The dataset paints a detailed picture of Medicare's financial and care responsibilities across a large and varied enrolee base.

Correlation of variables among high-throughput data

The initial analysis of impacts related to total days versus covered days revealed mean and frequency values that prompted further investigation into potential opportunities. To better understand the correlations in high-throughput data affecting LOS in healthcare settings, we conducted an additional analysis using a heatmap. 

One particularly significant finding was the strong positive correlation (r = 0.969) between total days of care and total program payments. This relationship indicates that more extended hospital stays are associated with higher healthcare costs. It highlights the importance of developing strategies for healthcare providers and policymakers to reduce unnecessary LOSs while maintaining quality care. In contrast, the study identifies negative correlations between total days of care and program payments per enrollee (r = -0.651). This situation implies a redistribution of costs across extended periods of care, underscoring the critical need for effective cost-management strategies. Such strategies must strive to balance the demands of financial sustainability with the imperative of delivering high-quality healthcare services. This dual focus is essential to foster a healthcare environment that is economically viable and capable of meeting patient needs.

**Figure 3:**
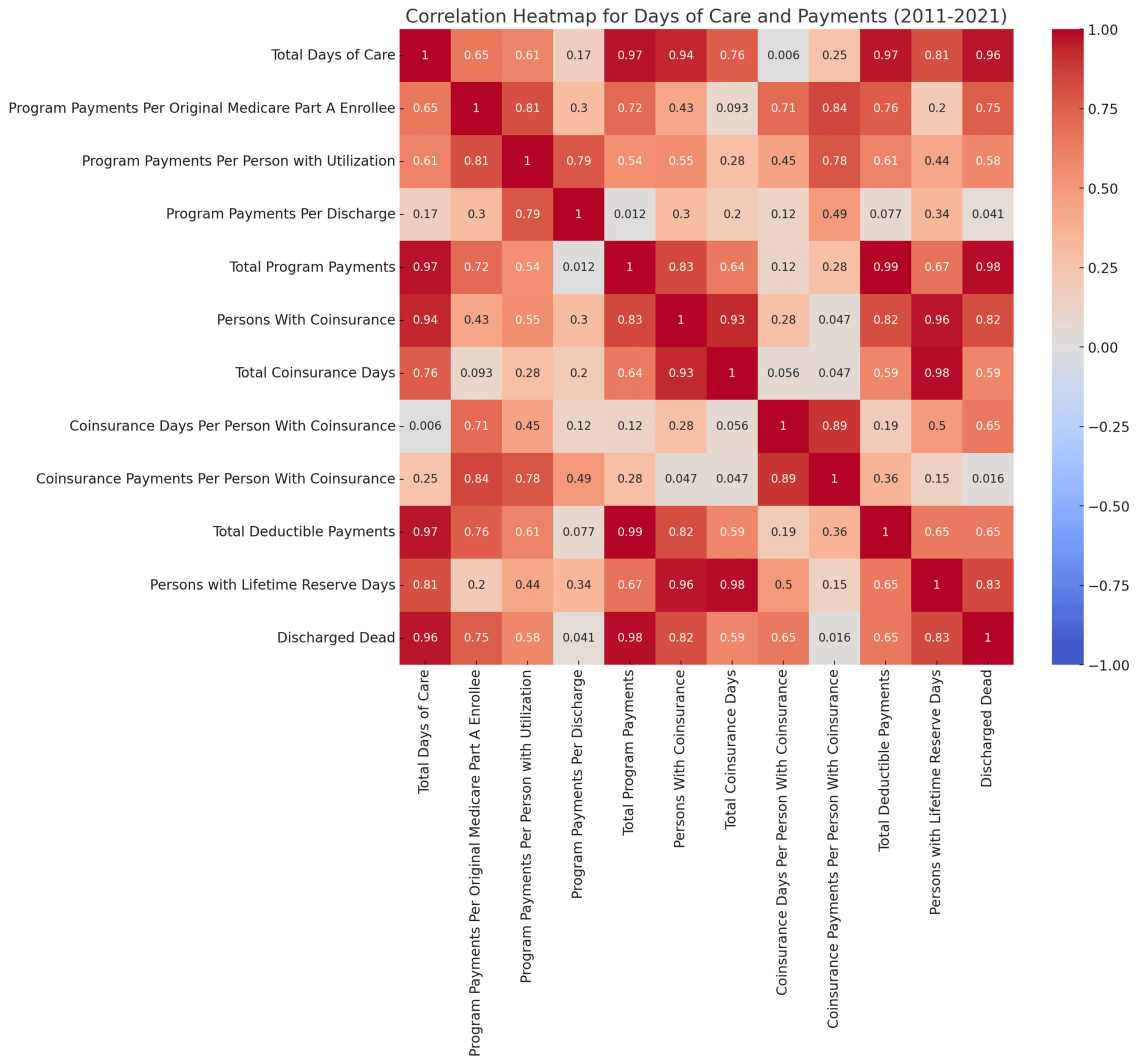
Correlation heatmap of healthcare metrics: days of care and payments (2011-2021) The image is a correlation heatmap visualizing the relationships between various healthcare metrics related to payments and days of care from 2011 to 2021. The axes represent different variables, such as "Total Days of Care," "Program Payments Per Original Medicare Part A Enrollee," and "Total Program Payments." The color scale ranges from dark red, indicating a strong positive correlation, to dark blue, indicating a strong negative correlation. Each cell contains a numerical value representing the correlation coefficient, ranging from -1 to 1, where values close to 1 indicate a strong positive correlation and those close to -1 indicate a strong negative correlation. For instance, "Total Days of Care" shows a strong positive correlation with "Total Program Payments" (0.97) and "Total Deductible Payments" (0.97), suggesting that, as one increases, the other tends to increase as well. This heatmap is a valuable tool for analyzing the interrelationships among healthcare metrics, which can inform policy decisions and healthcare management strategies.

Additionally, the analysis reveals that the interconnectedness of coinsurance-related variables sheds light on the influence of insurance frameworks on patient care. A strong positive correlation (0.935) between total days of care and the number of individuals with coinsurance signifies that extended hospitalizations often coincide with more patients requiring this form of coverage. Furthermore, this study presents trends that could enhance outcome prediction, exemplified by the high correlations between deceased patients' discharges and total days of care (0.963) and program payments (0.983). This knowledge allows healthcare organizations to identify at-risk populations and adopt targeted interventions. Using correlation values, we establish a quantitative basis for data-driven decision-making free from bias and avoid misleading outcomes. This approach empowers stakeholders to make informed choices that improve healthcare delivery, manage expenditures, and enhance patient outcomes across the healthcare system. Figure 3 shows that exploring the relationships among healthcare financial metrics, such as total days of care, program payments, and coinsurance metrics, clarifies the complex dynamics involved.

The correlation heatmap analysis identifies several statistically significant relationships among key healthcare payment and utilization metrics. Notably, a strong positive correlation (r = 0.97) exists between the total days of care and total program payments. This indicates a direct relationship, suggesting that longer durations of care are closely associated with higher overall payments. This finding implies that the length of care is a significant cost driver, potentially affecting payment structures across healthcare programs. Additionally, there is a relatively strong correlation (r = 0.96) between patients who are discharged deceased and total days of care. This correlation suggests that more extended hospital stays do not necessarily lead to better outcomes; instead, they may indicate a failure to address patients' needs adequately. The insights derived from this correlation emphasize that providing patients with the proper care at the right time should be a top priority for any hospital. The near-perfect correlation between total deductible payments and total program payments (r = 0.99) implies that variations in deductible amounts could substantially affect total healthcare expenditures. This highlights the importance of deductible structures in managing and forecasting healthcare costs. Other metrics exhibit moderately positive correlations, such as between persons with coinsurance and total coinsurance days (r = 0.76), program payments per discharge, and program payments per person with utilization (r = 0.79). These moderate correlations suggest an associative link, where higher utilization metrics correlate with increased program payments, though they may not be as dominant cost drivers as care duration and deductibles. Conversely, the negligible correlation observed between coinsurance days per person with coinsurance and total days of care (r = 0.006) suggests a little-to-no relationship, highlighting variability in how these metrics interact. Such weak correlations may indicate areas where other factors, beyond coinsurance and care duration, play more substantial roles.

These findings indicate strategic opportunities for healthcare providers and policymakers. Providers may benefit from focusing on care duration management to optimize reimbursement. For policymakers, the deductible structure is a significant consideration for controlling healthcare costs. To build on these insights, further analysis could benefit from segmenting data by demographics, payer types, or clinical conditions to understand underlying causative factors and tailor cost management strategies more precisely. Expanding upon these insights, it becomes evident that a nuanced approach to healthcare payment and utilization management could yield significant benefits. The strong correlations underscore potential areas of financial impact and suggest that these relationships may have underlying causes worthy of closer investigation. For instance, the high correlation between total days of care and program payments implies that resource allocation and care practices directly influence payment outcomes. This finding suggests that healthcare providers might consider adopting care protocols or LOS management practices aligning with clinical and financial goals.

Furthermore, the substantial correlation between deductible payments and program payments highlights the role of cost-sharing mechanisms in healthcare economics. Deductibles can act as both a financial burden and a regulatory tool, influencing patient behavior, access to services, and total program costs. By re-evaluating deductible structures, policymakers could address affordability issues while controlling the escalation of healthcare spending. This aligns with broader discussions on healthcare reform, where cost-sharing models are continually assessed for their impacts on utilization patterns and patient outcomes. The moderate correlations observed between persons with coinsurance, total coinsurance days, and utilization-based payment metrics suggest that while coinsurance does influence costs, it may be moderated by other factors such as patient demographics, type of care, or specific insurance policies. This indicates that policies focusing on coinsurance might have a localized effect rather than a broad impact on overall costs, emphasizing the need for targeted, data-informed adjustments to coinsurance structures. On the other hand, the lack of correlation between coinsurance days per person and total days of care suggests that coinsurance does not inherently extend or reduce care duration, implying that other determinants, such as care complexity, patient severity, or hospital policies, play more prominent roles in driving care length. This finding could guide policymakers and providers to examine alternative care aspects that may influence clinical outcomes and financial efficiency without directly altering coinsurance policies.

ARMC vs nationwide data

**Figure 4:**
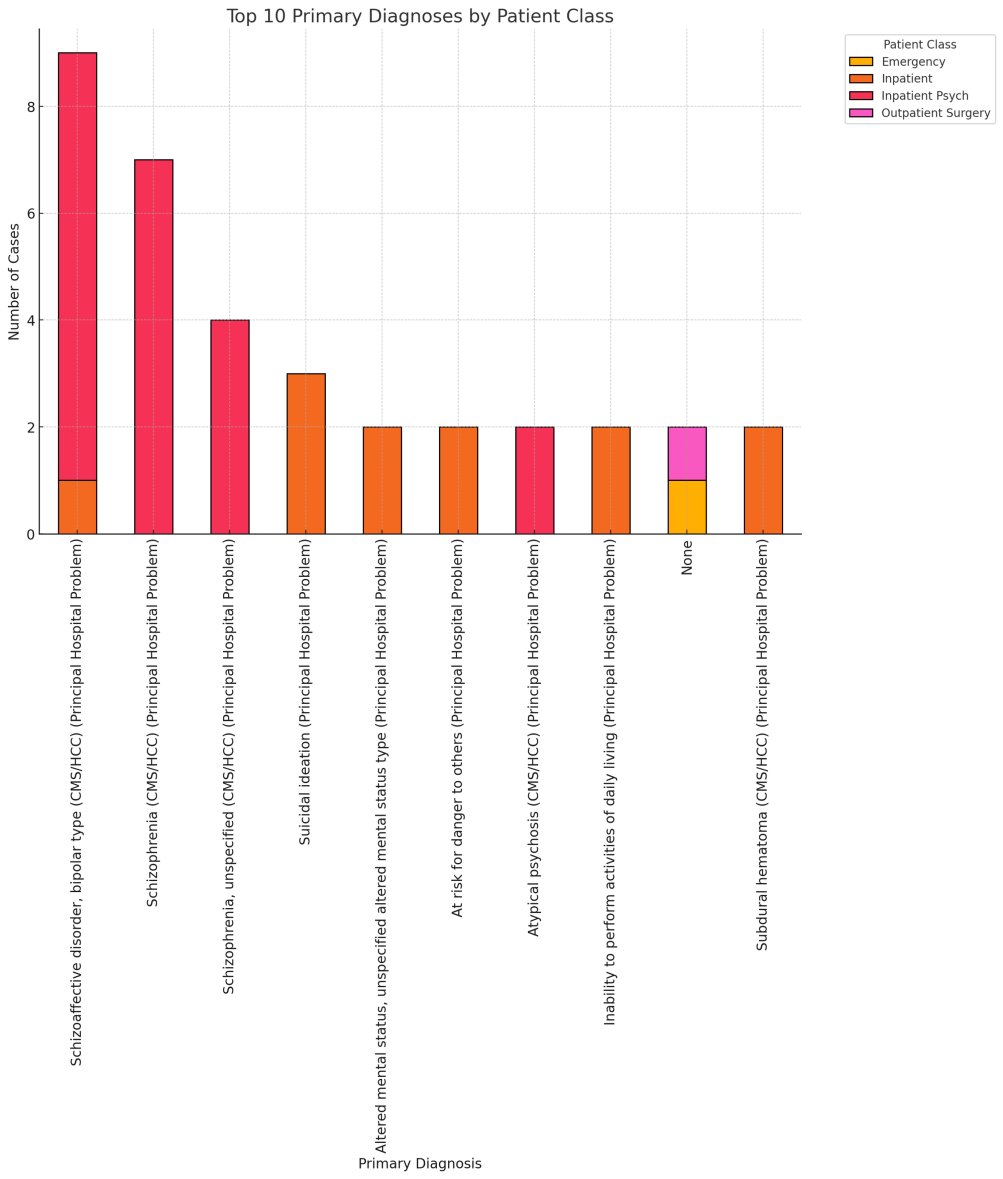
Top 10 primary/principal diagnoses by patient class This bar chart exhibits primary/principal diagnoses for patients with extended stays exceeding 18 days and their associated patient class (emergency, inpatient, inpatient psychiatry or outpatient surgery)

The study's findings reveal that the average LOS for patients admitted nationwide from 2011 to 2021 was around 5.7 days. It is essential to delve into extended stays that exceed this average, especially when taking a localized approach. We understood that analyzing data at the community level offers insight into specific health trends, demographic factors, and social determinants that might contribute to longer hospitalizations. This localized information can be pivotal in creating targeted interventions that address the community's unique needs. By comparing local data with national averages, healthcare providers can pinpoint areas needing improvement and set realistic performance benchmarks. Different regions may exhibit diverse patient populations with varying health requirements. Thus, having a comprehensive understanding of these characteristics is key for effectively tailoring care models to serve the community better, ultimately enhancing healthcare delivery and outcomes. As shown in Figure 4, based on data from patients presenting to ARMC between February 2022 and December 2024, our search identified 120 patients whose LOS ranged from 18 to 720 days. The bar chart exhibited in Figure 4 provides a detailed overview of the distribution of primary diagnoses across various patient classes: emergency, inpatient, inpatient psych, and outpatient surgery. Severe mental health conditions, such as schizoaffective disorder and schizophrenia, are notably prevalent in the Inpatient category, highlighting the significant challenges these disorders pose in terms of prolonged hospital stays at ARMC. The emergency category shows a moderate number of cases, particularly concerning acute mental health crises. In contrast, the outpatient surgery category reflects a significantly lower occurrence. These data emphasize the urgent requirement for enhanced mental health resources within hospital systems such as ARMC as the high prevalence of severe mental health diagnoses underscores the ongoing obstacles in adequately addressing mental healthcare.

Additionally, it is essential to recognize that extended stays often result from ambiguous clinical decision-making, where the guidelines for emergency or observation versus inpatient admission are not clearly defined or followed. Observation status is generally meant for patients who need short-term monitoring or treatment, typically under 24 hours. At the same time, observation status placement must be up to 48 hours, and inpatient admission is intended for patients requiring more intensive care than 48 hours, per the CMS two midnights rule [27]. When healthcare providers misinterpret a patient's condition or the criteria for these classifications, it can lead to inappropriate admissions, resulting in prolonged stays that may not align with the patient's actual clinical needs, a widespread patient throughput problem nationwide where bed management demands are escalating.

Adjusting confounders using a propensity score

Incorporating propensity scores into analyses of hospital utilization, LOS, and morbidity offers several substantial benefits [28]. Primarily, propensity scores adjust for confounding variables, such as patient co-morbidities and hospital quality, which might otherwise obscure the authentic relationships embedded in the data. For example, hospitals with poorer quality might show higher morbidity rates independent of patient characteristics, skewing the perception of outcomes. These factors are accounted for with propensity scores, enabling a more precise, more accurate assessment of patient outcomes. Moreover, propensity scores improve causal inference by estimating the likelihood of high morbidity based on observable characteristics, allowing for more precise comparisons between patients with similar scores. This comparison refines our understanding of how factors such as LOS or age potentially influence morbidity directly. Additionally, by addressing external variables that could affect hospital stay and morbidity rates, propensity scores significantly reduce selection bias, ensuring that analyses remain reliable. Consequently, they simplify complex relationships by condensing multiple confounding variables into a single metric more easily utilized in statistical models and visualizations.

**Figure 5:**
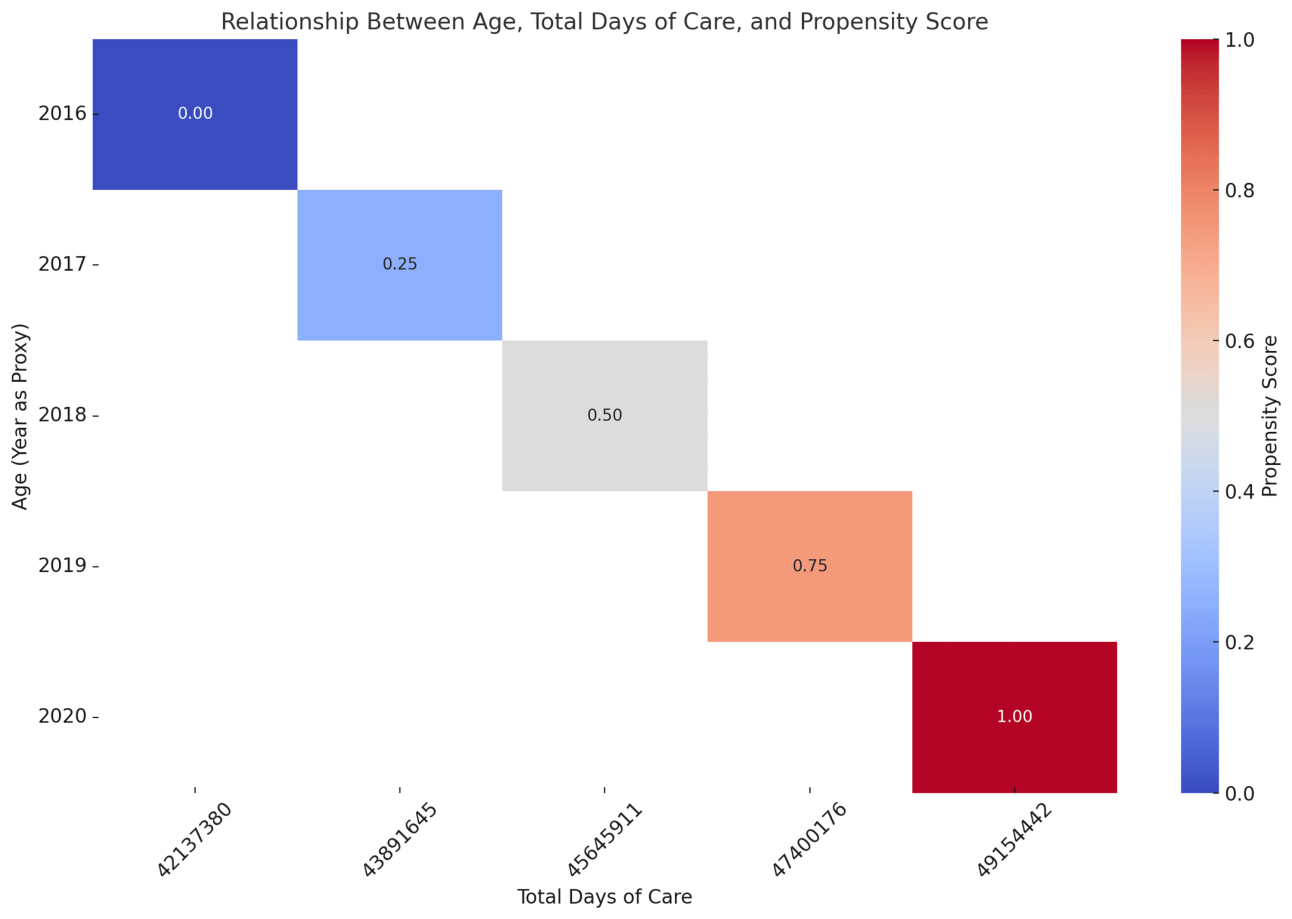
Adjusted morbidity score with propensity score

The practical application of propensity scores provides significant benefits, particularly in enhancing the clarity and effectiveness of data presentations through advanced visualization techniques. For example, data visualization methods supported by propensity scores, as shown in Figure 5, enable healthcare providers and researchers to observe adjusted relationships more clearly. This approach allows them to highlight critical trends or anomalies that may not be immediately visible, facilitating the identification of actionable factors influencing patient outcomes, especially among high-risk populations requiring targeted interventions. By delivering more profound insights into how irrelevant factors affect patient care and hospital operations, these analytical improvements support efforts to enhance healthcare delivery, improve patient-centered outcomes, and optimize the efficiency of HIE within hospitals. Moreover, in the realm of patient-centered care, utilizing propensity score adjustments aligns with the fundamental principles of personalized and equitable healthcare. By considering various patient characteristics and hospital contexts, this statistical tool helps identify high-risk groups that could benefit from specific interventions. For instance, hospitals can apply propensity score models to identify patients at a greater risk of adverse outcomes, facilitating the development of tailored care pathways that reduce morbidity and enhance overall patient satisfaction. This strategy improves the quality of care and ensures the efficient allocation of hospital resources, directing interventions where they are most needed.

Leveraging propensity scores in evaluating hospital care could lead to standardized, patient-centered performance metrics. By consistently accounting for confounding factors, propensity scores can support benchmarking hospital performance and strengthen quality improvement efforts nationally. Additionally, advancements in ML and big data analytics could further refine propensity score methodologies, allowing for real-time adjustments and predictions that enhance patient care strategies. Integrating propensity scores is crucial to creating a more equitable, data-driven healthcare system that prioritizes patient outcomes and experiences.

## Results

It is commonly assumed that a patient's LOS in a hospital directly correlates with the cost of care. However, our study provided evidence that clearly shows the relationship is more complex than it appears. The complexity of illnesses and nationwide data support this conclusion.

Cost of care: Longer hospital stays are generally associated with higher costs due to extended resource utilization. However, this relationship is not strictly linear and has directly impacted CMS Medicare spending. Factors such as care efficiency, hospital practices, and patient-specific needs can influence costs independently of LOS. Enrollment ranges from over 4.4 million to more than 32 million, averaging 18.7 million enrollees. Utilization metrics show that approximately 660,000 to 5.7 million individuals accessed services, with an average utilization of around 3.1 million. Total hospital discharges span from 1.1 million to 9.5 million, indicating diverse healthcare needs, and the total days of care range from 7.1 million to over 55 million, averaging 1,748 days per 1,000 enrollees. Financially, Medicare Part A expenditures range from $18.7 billion to over $112 billion, with an average cost of about $61 billion. The average costs per enrolee and user are $3,586 and $21,106, respectively, while the average coinsurance payments are around $9,296. The dataset illustrates Medicare Part A's complex financial and care responsibilities across its varied population. The analysis in Table 2 highlights the correlations between various healthcare metrics, revealing several key relationships. Total days of care show a strong positive correlation with total program payments (0.969) and the number of persons with coinsurance (0.935), indicating that longer care durations lead to higher spending and more individuals requiring coinsurance. However, a negative correlation exists between program payments per enrollee (-0.651) and per person with utilization (-0.608), suggesting that costs per enrollee decrease as care days increase.

Additionally, program payments exhibit a strong positive correlation between payments per original Medicare enrollee and person with utilization (0.811), while program payments per person with utilization and per discharge correlate closely (0.787). Total program payments correlate highly with total deductible payments (0.993) and total coinsurance days (0.635), demonstrating that higher overall spending often accompanies increased deductible payments and more coinsurance days. Furthermore, there is strong interconnectedness among coinsurance metrics, evidenced by a high correlation between total coinsurance days and the number of persons with coinsurance (0.928) and between coinsurance payments per person and days of coinsurance per individual (0.894). Finally, the number of discharges for patients who passed away correlates highly with total days of care (0.963) and total program payments (0.983), suggesting that more extended hospital stays and increased costs are linked to more severe health outcomes. The data indicate that increased care duration and total expenses are interconnected, with significant implications for coinsurance and health outcomes.

Hospital outcomes: The relationship between LOS and outcomes such as mortality and readmission rates is complex. Shorter stays might reduce exposure to hospital-acquired conditions but could also lead to premature discharges, increasing readmission risks. Conversely, extended stays might indicate complications or inadequate discharge planning. The results of this study align with previous research, such as those by Damery et al., which suggests that optimizing the LOS requires balancing efficient care delivery with comprehensive discharge processes to achieve positive outcomes [29]. About our findings, Table 2 provides a detailed analysis of the relationships among various hospital care metrics, focusing specifically on total days of care, covered days, and discharge outcomes across all Medicare Inpatient Hospitals and Medicare IPPS Short Stay Hospitals.

**Table 2:** Nationwide correlations among length of stay variables with respect to type of hospital ** Correlation is significant at the 0.01 level (2-tailed); * Correlation is significant at the 0.05 level (2-tailed).

Hospital Domain			Total Days of Care	Total Days of Care Per 1,000 Original Medicare Part A Enrolees	Total Days of Care Per Person with Utilization	Total Days of Care Per Discharge	Covered Days of Care	Covered Days of Care Per 1,000 Original Medicare Part A Enrolees	Covered Days of Care Per Person with Utilization	Covered Days of Care Per Discharge	Discharged Dead
All Medicare Inpatient Hospitals	Total Days of Care	Pearson Correlation	--								
		N	56								
	Total Days of Care Per 1,000 Original Medicare Part A Enrolees	Pearson Correlation	-0.452^**^	--							
		Sig. (2-tailed)	0.000								
		N	56	56							
	Total Days of Care Per Person with Utilization	Pearson Correlation	-0.624^**^	0.417^**^	--						
		Sig. (2-tailed)	0.000	0.001							
		N	56	56	56						
	Total Days of Care Per Discharge	Pearson Correlation	-0.530^**^	0.137	0.950^**^	--					
		Sig. (2-tailed)	0.000	0.314	0.000						
		N	56	56	56	56					
	Covered Days of Care	Pearson Correlation	0.999^**^	-0.444^**^	-0.637^**^	-0.547^**^	--				
		Sig. (2-tailed)	0.000	0.001	0.000	0.000					
		N	56	56	56	56	56				
	Covered Days of Care Per 1,000 Original Medicare Part A Enrollees	Pearson Correlation	-0.416^**^	0.996^**^	0.338^*^	0.052	-0.406^**^	--			
		Sig. (2-tailed)	0.001	0.000	0.011	0.705	0.002				
		N	56	56	56	56	56	56			
	Covered Days of Care Per Person with Utilization	Pearson Correlation	-0.632^**^	0.514^**^	0.989^**^	0.908^**^	-0.642^**^	0.441^**^	--		
		Sig. (2-tailed)	0.000	0.000	0.000	0.000	0.000	0.001			
		N	56	56	56	56	56	56	56		
	Covered Days of Care Per Discharge	Pearson Correlation	-0.488^**^	0.151	0.911^**^	0.962^**^	-0.500^**^	0.074	0.886^**^	--	
		Sig. (2-tailed)	0.000	0.265	0.000	0.000	0.000	0.587	0.000		
		N	56	56	56	56	56	56	56	56	
	Discharged Dead	Pearson Correlation	0.939^**^	-0.408^**^	-0.703^**^	-0.625^**^	0.950^**^	-0.349^**^	-0.689^**^	-0.540^**^	--
		Sig. (2-tailed)	0.000	0.002	0.000	0.000	0.000	0.010	0.000	0.000	
		N	54	54	54	54	54	54	54	54	54
Medicare IPPS Short Stay Hospitals	Total Days of Care	Pearson Correlation	--								
		N	56								
	Total Days of Care Per 1,000 Original Medicare Part A Enrolees	Pearson Correlation	-0.333^*^	--							
		Sig. (2-tailed)	0.012								
		N	56	56							
	Total Days of Care Per Person with Utilization	Pearson Correlation	-0.546^**^	0.452^**^	--						
		Sig. (2-tailed)	0.000	0.000							
		N	56	56	56						
	Total Days of Care Per Discharge	Pearson Correlation	-0.245	0.691^**^	0.325^*^	--					
		Sig. (2-tailed)	0.069	0.000	0.015						
		N	56	56	56	56					
	Covered Days of Care	Pearson Correlation	1.000^**^	-0.325^*^	-0.551^**^	-0.247	--				
		Sig. (2-tailed)	0.000	0.014	0.000	0.067					
		N	56	56	56	56	56				
	Covered Days of Care Per 1,000 Original Medicare Part A Enrolees	Pearson Correlation	-0.309^*^	0.995^**^	0.375^**^	0.662^**^	-0.300^*^	--			
		Sig. (2-tailed)	0.020	0.000	0.004	0.000	0.025				
		N	56	56	56	56	56	56			
	Covered Days of Care Per Person with Utilization	Pearson Correlation	-0.126	0.588^**^	0.015	0.943^**^	-0.125	0.584^**^	--		
		Sig. (2-tailed)	0.355	0.000	0.915	0.000	0.357	0.000			
		N	56	56	56	56	56	56	56		
	Covered Days of Care Per Discharge	Pearson Correlation	-0.186	0.690^**^	0.214	0.989^**^	-0.186	0.674^**^	0.967^**^	--	
		Sig. (2-tailed)	0.170	0.000	0.114	0.000	0.170	0.000	0.000		
		N	56	56	56	56	56	56	56	56	
	Discharged Dead	Pearson Correlation	0.756^**^	0.289^*^	-0.530^**^	0.381^**^	0.762^**^	0.301^*^	0.465^**^	0.439^**^	--
		Sig. (2-tailed)	0.000	0.044	0.000	0.007	0.000	0.036	0.001	0.002	
		N	49	49	49	49	49	49	49	49	49

In the All Medicare Inpatient Hospitals category, there is a nearly perfect positive correlation between total days of care and covered days of care, with a Pearson correlation of 0.999 (p < 0.01). This exceptionally close relationship indicates that, as hospitals increase the number of care days, nearly all of these are financially covered. Essentially, the greater the hospital utilization, the more care days align with insurance coverage, suggesting that high patient volumes are effectively backed by financial resources. However, total days of care have a moderate negative correlation with days per 1,000 enrollees (-0.452, p < 0.01) and per person with utilization (-0.624, p < 0.01). This pattern suggests that, while overall care days are high, the average days per individual enrolee or user are relatively low, indicating that resources are being distributed across a larger patient population, leading to less concentrated care on a per-person basis.

Another key insight is the strong positive correlation between total days of care and the number of patients discharged who are discharged dead, with a Pearson correlation of 0.939 (p < 0.01). This implies that hospitals with extended care days also tend to have higher discharge rates among deceased patients, suggesting that patients with more severe or complex health conditions are more likely to receive prolonged care. Similarly, covered days of care are strongly linked to discharged deaths (0.950, p < 0.01), indicating that, even when financial backing allows for extended stays, the critical nature of cases in these hospitals can lead to higher mortality rates, suggesting that extended care and coverage do not necessarily result in improved survival outcomes in severe cases.

Looking at the Medicare IPPS Short Stay Hospitals, there is again a high positive relationship between total days of care and covered days of care, with a Pearson correlation of 1.000 (p < 0.01), mirroring the trend in all Medicare hospitals and reinforcing that the greater total days of care also means high levels of insurance coverage. However, in contrast to the broader Medicare group, there is a positive correlation between total days per 1,000 enrolees and per person with utilization (0.452, p < 0.01) in short-stay hospitals. This correlation indicates that shorter, more intensive care is concentrated more heavily on individual patients, reflecting a different care approach in short-stay hospitals, where there is often more immediate and focused care per patient rather than spreading resources across the wider enrolee base.

Furthermore, in short-stay hospitals, the relationship between covered days of care and the number of patients discharged diseased is moderately strong, with a Pearson correlation of 0.762 (p < 0.01). This shows that higher coverage levels correlate with higher mortality at discharge even in these shorter-term settings. In both hospital types, higher coverage and longer stays are associated with increased mortality rates, potentially reflecting the severity of cases that require extended care and resources. Thus, while both extended care and financial backing are essential, they may also indicate the complex challenges faced by hospitals when dealing with critical and severe cases.

From the financial stratification above, we can see the value of linking financial incentives with care outcomes. While, from the general outlook, care seems to be equally distributed by Medicare nationwide, our results highlight the dire need for optimizing clinical efficiency via using evidence-based care pathways technology. The propensity score methodology for risk prediction proposed in this study proves to streamline the efficient utilization of resources without compromising outcomes. Additionally, since it is patient-centered, it can be scalable and integrated towards adopting a value-oriented care model, which will ultimately improve outcomes and reduce costs.

## Discussion

Limitations of the study

The study acknowledges several significant limitations that warrant consideration. First, it uses national data, which, while comprehensive, may exhibit variations in data collection methods across different hospitals. Furthermore, the generalizability of the results may be limited, particularly in rural or smaller healthcare settings that operate under distinct constraints. The analysis may also encounter challenges from confounding variables such as patient demographics, co-morbidities, and social determinants of health, which could influence LOS outcomes and complicate interpretations. Additionally, temporal limitations should be noted since the study may only partially capture shifts in healthcare policies, insurance practices, or technological advancements that can evolve over time and significantly affect LOS and care outcomes. Moreover, while the study focuses on quantitative data regarding LOS and care processes, it may only partially encompass qualitative elements such as patient experiences and satisfaction, which are vital for a holistic understanding of care effectiveness. The risks associated with HIE and cybersecurity breaches can also impact the relevance of findings related to data utilization and patient trust, given the rapid evolution of these factors in the healthcare landscape. Lastly, the dynamic nature of healthcare systems, including fluctuating reimbursement models and care delivery practices, highlights the importance of ongoing research to ensure insights remain relevant and actionable. By acknowledging these limitations, the study aims to present a balanced and nuanced understanding of hospital LOS and its broader implications, recognizing the intricate complexities that characterize healthcare delivery policies, insurance practices, or technological advancements that can evolve over time and significantly affect LOS and care outcomes. Moreover, while the study focuses on quantitative data regarding LOS and care processes, it may only partially encompass qualitative elements such as patient experiences and satisfaction, which are vital for a holistic understanding of care effectiveness. The risks associated with HIE and cybersecurity breaches can also impact the relevance of findings related to data utilization and patient trust, given the fast-paced evolution of these factors in the healthcare landscape. Lastly, the dynamic nature of healthcare systems, including fluctuating reimbursement models and care delivery practices, underscores the importance of ongoing research to ensure insights remain relevant and actionable. By acknowledging these limitations, the study aims to present a balanced and nuanced understanding of hospital LOS and its broader implications, recognizing the intricate complexities that characterize healthcare delivery.

Future applications

In contemporary Medicine, the integration of generative artificial intelligence (AI) presents a critical opportunity for establishing a collaborative data matrix focused on optimizing HIE and improving the quality of hospital care. A key challenge providers and progressive healthcare technologists face is ensuring the privacy and security of extramural HIEs. Our study has investigated the utilization of propensity scores to mitigate confounding factors, thereby enabling accurate assessment of patient acuity and satisfaction while mitigating undesired ML outputs. The objective is to address the biased clinical medical decision-making induced by AI, among other ethical considerations addressed in many studies, such as that of MacIntyre et al. [24]. The results demonstrated that the scalability of propensity score induction onto co-pilot ML supports patient-centered care and outcomes and enhances HIE maneuvers. HIE refers to the secure and standardized method of sharing and accessing patient health information across various healthcare organizations, such as hospitals, clinics, pharmacies, and labs. It allows healthcare providers, patients, and other stakeholders to electronically access and exchange medical data to improve the quality, safety, and efficiency of healthcare delivery. The goal of HIE is to create a seamless flow of health information across systems, ensuring that providers have the most up-to-date and comprehensive patient data for informed decision-making, reducing redundant tests, preventing medical errors, and enhancing care coordination. HIE may operate regionally, statewide, or nationally, and is a vital component of modern healthcare infrastructure, especially in supporting transitions of care and population health management.

There is an increasing potential to combine propensity score analysis with blockchain technology to facilitate secure data dissemination through decentralized ledgers, enabling hospitals to share data while safeguarding the integrity of patient information. Implementing smart contracts within blockchain frameworks further enhances the efficiency of data sharing and governance. These self-executing agreements, with terms coded directly into software, streamline compliance with ethical and regulatory standards such as HIPAA and GDPR. For example, a smart contract could stipulate precise access rules to data based on purpose and conditions, effectively removing intermediaries and alleviating administrative burdens. The concept of patients arriving at the emergency department (ED) with their complete prior medical information readily accessible is a transformative proposition for healthcare. Using blockchain technology, patient data from previous visits, including diagnoses, treatments, medications, and diagnostic results, could be securely stored and accessed in real time upon arrival. This seamless transfer of information would eliminate the redundancies and inefficiencies often associated with ED workflows, where incomplete or inaccessible medical histories delay accurate diagnosis and treatment. This will ultimately also improve ED quality measures such as OP-18 and OP-21. Blockchain’s decentralization and immutability ensure that the data are correct, unaltered, and readily available, fostering a continuity of care that improves decision-making and patient outcomes.

Integrating blockchain-supported data systems with propensity score methodologies further enhances ED operations, particularly in distinguishing between patients who may benefit from observation versus inpatient admission. Propensity score models, applied in real-time to the patient’s comprehensive history, could predict the likelihood of outcomes such as readmission risk or the need for critical interventions. For example, ED physicians could use these models to identify patients who can be safely monitored in observation units, optimizing bed usage and reducing unnecessary inpatient admissions. This capability minimizes healthcare costs and aligns with quality metrics tied to the LOS and 30-day readmission rates, which are critical for hospital performance under value-based care models. The impact on quality metrics is substantial, as the availability of accurate, blockchain-secured data ensures more precise documentation, coding, and resource utilization. Improved documentation directly affects hospital ratings, such as CMS star ratings and leapfrog scores, which rely heavily on metrics such as LOS, mortality rates, and patient safety indicators.

Furthermore, by reducing delays in treatment and ensuring appropriate triage decisions, ED throughput is enhanced, contributing to better patient satisfaction scores and more efficient resource allocation. Integrating blockchain technology with real-time analytics and propensity score models bridges the gap between data availability and actionable insights, fundamentally reshaping how the ED functions as a critical gateway to inpatient care and beyond. This is particularly vital in multi-institutional settings, where providers often need help with consistent data-sharing agreements and interoperability challenges. Blockchain technology ensures that data utilized for propensity score analysis remains uniform, anonymized when necessary, and accessible solely to authorized parties. One prevalent issue within EHR systems is the confusion caused by order sets that are modified, reinstated, or deleted post-discharge. The application of blockchain can enhance the immutability of records, guaranteeing the integrity of data once it is entered. This characteristic is essential for propensity score applications, as even minor dataset alterations could skew model validity, undermining the accuracy of patient acuity assessments.

The fidelity of information derived from these advances can rectify erroneous predictions related to GMLOS, which have historically impeded providers' ability to make informed medical necessity decisions, ultimately affecting care delivery. Our findings reveal that an exclusive focus on EHR systems predicting LOS targets can prematurely deplete insurance benefits and impede equitable fund allocation at national and local patient levels. Consequently, a healthcare paradigm that embraces innovative resource utilization approaches will enhance patient experience and hospital efficiency. A central emphasis in this endeavor is the exploration and implementation of EHR solutions coupled with managed care interventions to optimize patient outcomes. 

The inadequacies of a one-size-fits-all predictive modeling approach highlight the necessity for a rigorous examination of the metrics employed in healthcare delivery. This critical assessment is vital to ensure that these metrics align with the overarching objective of enhancing patient care and improving operational efficiency. Patient outcomes are essential indicators for evaluating the effectiveness of healthcare delivery systems. Metrics that include functional recovery, long-term survival rates, and patient-reported outcomes offer a more comprehensive understanding of healthcare quality. However, it is disconcerting that these pivotal metrics often remain excluded from managed care guidelines, further entrenching the focus on LOS. Incorporating outcome-based measures, such as the propensity scoring mechanisms discussed in our research, into managed care policies would create a more equitable approach aligned with value-based care principles. Moreover, our study underscores the significant effects of national patients’ socioeconomic status on enrollment patterns, hospital-covered days, and the ramifications of demographic factors, such as age, on healthcare needs and outcomes.

## Conclusions

Since we encountered many evolving health events, such as the COVID-19 pandemic, developing tools that empower patients' decisions must consider a framework of ML models that integrate variables and tailor insights prospectively based on individual patient characteristics. Improving digital tools that meet this vision is possible and goes beyond just calculating relative weights of diagnoses and grouping illnesses. Integrating blockchain and propensity score analytics in hospital workflows significantly improves upon the current DRG system, which often falls short in addressing the complexities of modern healthcare delivery. The DRG system assigns payments based on predetermined categories that reflect the patient’s primary diagnosis, procedures, and associated resource use. While effective in standardizing payments, it struggles with real-time adaptability, nuanced clinical decision-making, and comprehensive data integration. By contrast, blockchain enables secure, instant access to a patient’s medical history across institutions, while propensity score analytics provide predictive insights that tailor care pathways to individual patient needs. This dual approach allows for more precise resource allocation and better alignment of treatment with clinical outcomes, reducing inefficiencies inherent in the DRG system. The DRG system is retrospective and relies heavily on accurate documentation and coding afterward. This reliance can create gaps in real-time decision support and pose challenges in ensuring that documentation accurately reflects the severity of illness (SOI) or risk of mortality (ROM). Blockchain technology addresses these shortcomings by guaranteeing immutable and real-time data availability, which improves documentation accuracy at the point of care. Additionally, propensity score analytics enhance predictive decision-making, providing dynamic guidance on whether a patient should be admitted, placed under observation, or safely discharged. For instance, while the DRG system may categorize patients with chest pain under a broad cardiac-related DRG, propensity score models provide individualized risk stratification that supports more tailored care. This method reduces unnecessary admissions and ensures appropriate billing by aligning resource use with clinical needs.

Our study has also observed that propensity scores are scalable and have prospects to strengthen HIE in ways the DRG system cannot. While the DRG's GMLOS system indirectly incentivizes hospitals to reduce LOS and readmissions, it lacks mechanisms to ensure these outcomes result from optimal clinical care rather than cost-cutting measures. However, blockchain-secured data and propensity score analytics provide transparency and predictive power to improve 30-day readmission rates, patient safety indicators, and CMS star ratings. For example, real-time access to historical data ensures appropriate triage decisions, reducing preventable readmissions, while blockchain’s immutability supports audit readiness and compliance with value-based care models. In essence, this combined approach transcends the static and retrospective nature of DRGs, delivering real-time, patient-centered solutions that enhance clinical outcomes, operational efficiency, and financial sustainability. Since patients are the main focus of our study, creating engaging, educational, and user-friendly digital tools is necessary to encourage them to take an active role in their care delivery. Additionally, these tools must guide healthcare providers without leading to biased medical decision-making or compromising HIPAA practices. Patient portals promote preventive health actions, such as vaccinations, screenings, and lifestyle changes, which can help reduce the risk of chronic diseases and minimize costly treatments. The proposed methods of assessing LOS and medical decision-making MDM place information obtained from patient histories of illnesses in a position of readiness to predict outcomes and inform risk stratification and cumulative nationwide health trends. This shift in focus necessitates a reevaluation of healthcare providers' priorities, moving from merely minimizing hospital stays to emphasizing patient-centered outcomes. This change can enhance provider job satisfaction and improve the quality of care delivered. Insurers are likely to benefit from adopting more sustainable financial models that reward long-term health improvements, in contrast to traditional models that prioritize short-term metrics. From the patient’s perspective, a personalized approach to care that considers individual socioeconomic factors and overall well-being is expected to lead to better health outcomes and greater satisfaction with the healthcare experience. Additionally, policymakers will gain access to data-driven insights from comprehensive and integrated metrics, facilitating informed decision-making and promoting equity and efficiency within the healthcare system. Propelling informed collaboration among diverse stakeholders, including clinicians, administrators, and patient-managed care, can transform into an inclusive framework that effectively meets our population's complex, multifaceted needs. This expansion will lead to improved healthcare delivery and outcomes, but it requires a national commitment to exploring new pathways driven by research and visionary thinking.

## Appendices

**Table 3:** Arrowhead Regional Medical Center ARMC data for patients exceeding 18 days length of stay (February 2022-December 2024) This report covers patients admitted to ARMC between February 2022 and December 2024, detailing their length of hospital stay, which varies from 18 days to 730 days. It highlights the patients' primary diagnoses, length of stay (in days and hours), patient classification, and both primary and secondary insurance coverages.

Primary Problem	Length of Stay	Patient Class	Primary Cvg Payer	Pat Secondary CVG Payer Name
Cerebral venous sinus thrombosis (Principal Hospital Problem)	17d 23h	Inpatient	MEDICARE	INLAND EMPIRE HEALTH PLAN MEDI-CAL MANAGED
Renal rupture, right, initial encounter (Principal Hospital Problem)	18d 10h	Inpatient	MEDICARE	INLAND EMPIRE HEALTH PLAN MEDI-CAL MANAGED
Chronic bilateral low back pain, unspecified whether sciatica present (Principal Hospital Problem)	17d 3h	Inpatient	MEDICARE	INLAND EMPIRE HEALTH PLAN MEDI-CAL MANAGED
Intraparenchymal hemorrhage of brain (CMS/HCC) (Principal Hospital Problem)	17d 20h	Inpatient	MEDI-CAL	
Suicidal ideations (Principal Hospital Problem)	18d 12h	Inpatient	MOLINA MANAGED MEDICARE	MOLINA MEDICAID
Colon cancer high risk (Principal Hospital Problem)	19d 1h	Inpatient	INLAND EMPIRE HEALTH PLAN MEDI-CAL MANAGED	
Cellulitis of right foot (Principal Hospital Problem)	18d 19h	Inpatient	LA CARE HEALTH PLAN	
Atypical psychosis (CMS/HCC) (Principal Hospital Problem)	18d 17h	Inpatient Psych	MEDICARE	
Trauma (Principal Hospital Problem)	19d 4h	Inpatient	INLAND EMPIRE HEALTH PLAN MEDI-CAL MANAGED	
Problem with vascular access (Principal Hospital Problem)	19d 9h	Inpatient	MEDICARE	INLAND EMPIRE HEALTH PLAN MEDI-CAL MANAGED
Subarachnoid bleed (CMS/HCC) (Principal Hospital Problem)	19d 22h	Inpatient	BCBS CA	
Intracerebral hemorrhage, nontraumatic (CMS/HCC) (Principal Hospital Problem)	20d 6h	Inpatient	INLAND EMPIRE HEALTH PLAN MEDI-CAL MANAGED	
Biventricular heart failure (CMS/HCC) (Principal Hospital Problem)	20d 23h	Inpatient	MEDI-CAL	
Squamous cell carcinoma of vocal cord (CMS/HCC) (Principal Hospital Problem)	20d 18h	Inpatient	MEDICARE	MEDI-CAL
Stroke-like symptoms (Principal Hospital Problem)	21d 19h	Inpatient	BLUE CROSS MEDI-CAL	
Acute pulmonary embolism (CMS/HCC) (Principal Hospital Problem)	21d 19h	Inpatient	MOLINA MEDICAID	
Tibial fracture (Additional Hospital Problems)	23d 11h	Inpatient	INLAND EMPIRE HEALTH PLAN MEDI-CAL MANAGED	
Suicidal ideation (Principal Hospital Problem)	22d 16h	Inpatient	INLAND EMPIRE HEALTH PLAN MEDI-CAL MANAGED	
Infection and inflammatory reaction due to internal right knee prosthesis, initial encounter (CMS/HCC) (Principal Hospital Problem)	23d 11h	Inpatient	UNITED HEALTHCARE MEDICARE	
Intraparenchymal hematoma of brain (CMS/HCC) (Principal Hospital Problem)	23d 7h	Inpatient	INLAND EMPIRE HEALTH PLAN MEDI-CAL MANAGED	
Hypertension	22d 13h	Inpatient	OCD SHERIFF PAYOR	
Gastrointestinal hemorrhage, unspecified gastrointestinal hemorrhage type (Principal Hospital Problem)	24d 6h	Inpatient	MEDICARE	INLAND EMPIRE HEALTH PLAN MEDI-CAL MANAGED
Schizoaffective disorder, bipolar type (CMS/HCC) (Principal Hospital Problem)	24d 10h	Inpatient Psych	MEDICARE	INLAND EMPIRE HEALTH PLAN MEDI-CAL MANAGED
Sickle cell anemia (CMS/HCC) (Principal Hospital Problem)	24d 18h	Inpatient	INLAND EMPIRE HEALTH PLAN MANAGED MEDICARE	
Acute encephalopathy (Principal Hospital Problem)	24d 18h	Inpatient	MEDI-CAL	
Bilateral pleural effusion (Principal Hospital Problem)	25d 23h	Inpatient	ALIGNMENT HEALTHCARE	INLAND EMPIRE HEALTH PLAN MEDI-CAL MANAGED
Patient needs psychiatric hold for evaluation (Principal Hospital Problem)	27d 6h	Inpatient	AETNA MEDICARE	
Schizoaffective disorder (CMS/HCC) (Principal Hospital Problem)	27d 15h	Inpatient Psych	MEDI-CAL	
Subdural hematoma (CMS/HCC) (Principal Hospital Problem)	27d 14h	Inpatient	BLUE CROSS	
Necrotizing fasciitis (CMS/HCC) (Principal Hospital Problem)	29d 8h	Inpatient	MEDICARE	INLAND EMPIRE HEALTH PLAN MEDI-CAL MANAGED
Hepatic encephalopathy (CMS/HCC) (Principal Hospital Problem)	29d 12h	Inpatient	INLAND EMPIRE HEALTH PLAN MEDI-CAL MANAGED	
Neck mass (Principal Hospital Problem)	30d 12h	Inpatient	UNITED HEALTHCARE MEDICARE	
Osteomyelitis (CMS/HCC) (Principal Hospital Problem)	31d 15h	Inpatient	MEDI-CAL ACA (INPATIENT)	
Schizoaffective disorder, bipolar type (CMS/HCC) (Principal Hospital Problem)	31d 12h	Inpatient Psych	INLAND EMPIRE HEALTH PLAN MEDI-CAL MANAGED	
Severe sepsis (CMS/HCC) (Principal Hospital Problem)	33d 6h	Inpatient	INLAND EMPIRE HEALTH PLAN MEDI-CAL MANAGED	
Suicidal ideation (Principal Hospital Problem)	32d 17h	Inpatient	MEDICARE	MEDI-CAL
Vertebral osteomyelitis (CMS/HCC) (Principal Hospital Problem)	34d 13h	Inpatient	MOLINA MEDICAID	
Cholecystitis (Principal Hospital Problem)	34d 13h	Inpatient	MEDI-CAL	
Dissection of abdominal aorta (CMS/HCC) (Principal Hospital Problem)	38d 7h	Inpatient	BLUE CROSS MEDI-CAL	
SIRS (systemic inflammatory response syndrome) (CMS/HCC) (Principal Hospital Problem)	38d 16h	Inpatient	MEDICARE	INLAND EMPIRE HEALTH PLAN MEDI-CAL MANAGED
Schizoaffective disorder, unspecified type (CMS/HCC) (Principal Hospital Problem)	38d 23h	Inpatient Psych	INLAND EMPIRE HEALTH PLAN MEDI-CAL MANAGED	
ESRD (end stage renal disease) (CMS/HCC) (Additional Hospital Problems)	39d 20h	Inpatient	INLAND EMPIRE HEALTH PLAN MANAGED MEDICARE	
Schizoaffective disorder, bipolar type (CMS/HCC) (Principal Hospital Problem)	40d 2h	Inpatient	INLAND EMPIRE HEALTH PLAN MEDI-CAL MANAGED	
Schizophrenia (CMS/HCC) (Principal Hospital Problem)	39d 22h	Inpatient Psych	INLAND EMPIRE HEALTH PLAN MEDI-CAL MANAGED	
Schizoaffective disorder, bipolar type (CMS/HCC) (Principal Hospital Problem)	41d	Inpatient Psych	INLAND EMPIRE HEALTH PLAN MEDI-CAL MANAGED	
Schizophrenia (CMS/HCC) (Principal Hospital Problem)	41d	Inpatient Psych	INLAND EMPIRE HEALTH PLAN MEDI-CAL MANAGED	
Acute respiratory failure (CMS/HCC) (Principal Hospital Problem)	42d 19h	Inpatient	MEDI-CAL ACA (INPATIENT)	
Schizophrenia (CMS/HCC) (Principal Hospital Problem)	43d 15h	Inpatient Psych	INLAND EMPIRE HEALTH PLAN MEDI-CAL MANAGED	
Osteomyelitis of left foot (CMS/HCC) (Principal Hospital Problem)	43d 21h	Inpatient	MEDI-CAL ACA (INPATIENT)	
Cholangitis (Principal Hospital Problem)	44d 22h	Inpatient	UNITED HEALTHCARE	
Inability to perform activities of daily living (Principal Hospital Problem)	44d 23h	Inpatient	MEDICARE	MEDI-CAL
Schizoaffective disorder, bipolar type (CMS/HCC) (Principal Hospital Problem)	45d 23h	Inpatient Psych		
Baby premature 32 weeks (Principal Hospital Problem)	47d 2h	Inpatient	MEDI-CAL	MOLINA MEDICAID
Adenocarcinoma of lung (CMS/HCC) (Principal Hospital Problem)	50d 4h	Inpatient	MEDI-CAL ACA (INPATIENT)	
Acute hypoxic respiratory failure (CMS/HCC) (Principal Hospital Problem)	52d 4h	Inpatient	INLAND EMPIRE HEALTH PLAN MEDI-CAL MANAGED	
Respiratory distress, acute (Principal Hospital Problem)	52d 16h	Inpatient	INLAND EMPIRE HEALTH PLAN MEDI-CAL MANAGED	
Subdural hematoma (CMS/HCC) (Principal Hospital Problem)	52d 19h	Inpatient	UNITED HEALTHCARE MEDICARE	MEDI-CAL
Altered mental status, unspecified altered mental status type (Principal Hospital Problem)	52d 20h	Inpatient	AETNA	
Bilateral lower extremity edema (Principal Hospital Problem)	53d 12h	Inpatient	BLUE CROSS MEDICARE	INLAND EMPIRE HEALTH PLAN MEDI-CAL MANAGED
Schizophrenia, unspecified (CMS/HCC) (Principal Hospital Problem)	55d 23h	Inpatient Psych	INLAND EMPIRE HEALTH PLAN MEDI-CAL MANAGED	
Gangrene of toe of both feet (CMS/HCC) (Principal Hospital Problem)	59d 14h	Inpatient	MEDICARE	INLAND EMPIRE HEALTH PLAN MANAGED MEDICARE
SDH (subdural hematoma) (CMS/HCC) (Principal Hospital Problem)	62d 23h	Inpatient	INLAND EMPIRE HEALTH PLAN MEDI-CAL MANAGED	
Recurrent falls (Principal Hospital Problem)	64d 4h	Inpatient	BLUE CROSS MEDICARE	KAISER PERMANENTE MEDI-CAL
Sepsis (CMS/HCC) (Principal Hospital Problem)	65d 1h	Inpatient	HEALTH NET MEDI-CAL	
Skin ulcer of sacrum with necrosis of muscle (CMS/HCC) (Principal Hospital Problem)	72d 23h	Inpatient	MOLINA MEDICAID	
Fall (Principal Hospital Problem)	73d 11h	Inpatient	AETNA MEDICARE	BLUE CROSS
Schizophrenia, unspecified (CMS/HCC) (Principal Hospital Problem)	73d 17h	Inpatient Psych	MEDI-CAL	
Schizoaffective disorder, bipolar type (CMS/HCC) (Principal Hospital Problem)	73d 23h	Inpatient Psych	MEDICARE	
Generalized weakness (Principal Hospital Problem)	75d 16h	Inpatient	INNOVAGE	INNOVAGE MEDICAID
Renal mass (Principal Hospital Problem)	77d 21h	Inpatient	MEDI-CAL ACA (INPATIENT)	
MVA (motor vehicle accident), initial encounter (Principal Hospital Problem)	80d 10h	Inpatient	LA CARE HEALTH PLAN	
Burn (any degree) involving 40-49% of body surface (Principal Hospital Problem)	82d 18h	Inpatient	HEALTH NET MEDI-CAL	
Bipolar disorder, unspecified (CMS/HCC) (Principal Hospital Problem)	82d 22h	Inpatient Psych	INLAND EMPIRE HEALTH PLAN MEDI-CAL MANAGED	
At risk for danger to others (Principal Hospital Problem)	85d 10h	Inpatient	SCAN MEDICARE	INLAND EMPIRE HEALTH PLAN MEDI-CAL MANAGED
Atypical psychosis (CMS/HCC) (Principal Hospital Problem)	84d 14h	Inpatient Psych	INLAND EMPIRE HEALTH PLAN MEDI-CAL MANAGED	
Weakness (Principal Hospital Problem)	88d 4h	Inpatient	INLAND EMPIRE HEALTH PLAN MEDI-CAL MANAGED	
Schizoaffective disorder, bipolar type (CMS/HCC) (Principal Hospital Problem)	89d	Inpatient Psych	INLAND EMPIRE HEALTH PLAN MEDI-CAL MANAGED	
Housing insecurity (Principal Hospital Problem)	89d 22h	Inpatient	INLAND EMPIRE HEALTH PLAN MANAGED MEDICARE	
Psychosis, unspecified psychosis type (CMS/HCC) (Principal Hospital Problem)	93d 2h	Inpatient Psych	INLAND EMPIRE HEALTH PLAN MEDI-CAL MANAGED	
At risk for danger to others (Principal Hospital Problem)	94d 14h	Inpatient	MEDICARE	INLAND EMPIRE HEALTH PLAN MEDI-CAL MANAGED
Schizophrenia (CMS/HCC) (Principal Hospital Problem)	97d 1h	Inpatient Psych		
Suicidal ideation (Principal Hospital Problem)	97d 21h	Inpatient	MEDICARE	INLAND EMPIRE HEALTH PLAN MEDI-CAL MANAGED
Schizoaffective disorder, unspecified condition (CMS/HCC) (Principal Hospital Problem)	102d 18h	Inpatient Psych		
Unable to care for self (Principal Hospital Problem)	104d 14h	Inpatient	BLUE CROSS MEDICARE	GOLD COAST HEALTH PLAN
Inability to perform activities of daily living (Principal Hospital Problem)	106d 12h	Inpatient	HUMANA MEDICARE ADVANTAGE	
Discitis of multiple sites of spine (Principal Hospital Problem)	110d 23h	Inpatient	INLAND EMPIRE HEALTH PLAN MEDI-CAL MANAGED	
Bipolar 1 disorder (CMS/HCC) (Principal Hospital Problem)	112d 16h	Inpatient	INLAND EMPIRE HEALTH PLAN MANAGED MEDICARE	
Schizophrenia (CMS/HCC) (Principal Hospital Problem)	114d 17h	Inpatient Psych	INLAND EMPIRE HEALTH PLAN MEDI-CAL MANAGED	
Delirium with dementia (CMS/HCC) (Principal Hospital Problem)	116d 22h	Inpatient	INLAND EMPIRE HEALTH PLAN MANAGED MEDICARE	
Schizoaffective disorder, unspecified (CMS/HCC) (Principal Hospital Problem)	116d 17h	Inpatient Psych	MEDICARE	MEDI-CAL
Mass, brain (Principal Hospital Problem)	124d 14h	Inpatient	UNITED HEALTHCARE MEDICARE	
Aggression (Principal Hospital Problem)	134d 19h	Inpatient	MEDI-CAL ACA (INPATIENT)	
Altered mental status, unspecified altered mental status type (Principal Hospital Problem)	135d 20h	Inpatient	INLAND EMPIRE HEALTH PLAN MEDI-CAL MANAGED	MEDI-CAL
Schizophrenia (CMS/HCC) (Principal Hospital Problem)	136d 18h	Inpatient Psych	INLAND EMPIRE HEALTH PLAN MEDI-CAL MANAGED	
Syncope (Principal Hospital Problem)	138d 8h	Inpatient	MEDI-CAL	MOLINA MEDICAID
Trauma (Principal Hospital Problem)	157d 13h	Inpatient		
Acute hypoxemic respiratory failure (CMS/HCC) (Principal Hospital Problem)	158d 8h	Inpatient	MEDICARE	INLAND EMPIRE HEALTH PLAN MEDI-CAL MANAGED
Hyponatremia (Principal Hospital Problem)	191d 6h	Inpatient	UNITED HEALTHCARE MEDICARE	MEDI-CAL
Schizophrenia, unspecified (CMS/HCC) (Principal Hospital Problem)	208d 16h	Inpatient Psych	MEDICARE	INLAND EMPIRE HEALTH PLAN MEDI-CAL MANAGED
AKI (acute kidney injury) (CMS/HCC) (Principal Hospital Problem)	211d 20h	Inpatient	INLAND EMPIRE HEALTH PLAN MEDI-CAL MANAGED	INNOVAGE
Schizoaffective disorder, chronic condition with acute exacerbation (CMS/HCC) (Principal Hospital Problem)	214d 16h	Inpatient Psych	MEDI-CAL ACA (INPATIENT)	
Schizoaffective disorder, bipolar type (CMS/HCC) (Principal Hospital Problem)	239d 15h	Inpatient Psych	MEDICARE	
Squamous cell carcinoma of maxillary sinus (CMS/HCC) (Principal Hospital Problem)	240d 13h	Inpatient	INLAND EMPIRE HEALTH PLAN MEDI-CAL MANAGED	MEDI-CAL
Epilepsy (CMS/HCC) (Principal Hospital Problem)	253d 19h	Inpatient	BLUE CROSS MEDI-CAL	OCD SHERIFF PAYOR
Disseminated coccidioidomycosis (Principal Hospital Problem)	258d 15h	Inpatient	INLAND EMPIRE HEALTH PLAN MEDI-CAL MANAGED	
Other dysphagia (Principal Hospital Problem)	269d 7h	Inpatient	MEDICARE	
Dementia associated with other underlying disease, unspecified dementia severity, unspecified whether behavioral, psychotic, or mood disturbance or anxiety (CMS/HCC) (Principal Hospital Problem)	270d 1h	Inpatient	VETERANS ADMINISTRATION	MEDICARE
Schizoaffective disorder, bipolar type (CMS/HCC) (Principal Hospital Problem)	332d 16h	Inpatient Psych	MEDICARE	
Schizophrenia, unspecified (CMS/HCC) (Principal Hospital Problem)	361d 22h	Inpatient Psych	MEDICARE	
Schizophrenia (CMS/HCC) (Principal Hospital Problem)	425d 15h	Inpatient Psych	MEDI-CAL	
Cerebral palsy, unspecified type (CMS/HCC) (Principal Hospital Problem)	441d 19h	Inpatient	OCD SHERIFF PAYOR	MEDICARE
Chronic intestinal pseudo-obstruction (Principal Hospital Problem)	554d 14h	Inpatient	INLAND EMPIRE HEALTH PLAN MEDI-CAL MANAGED	
Paranoid schizophrenia (CMS/HCC) (Principal Hospital Problem)	730d 13h	Inpatient Psych	INLAND EMPIRE HEALTH PLAN MEDI-CAL MANAGED	
